# DESTINY-Breast08: A Phase Ib Study of Trastuzumab Deruxtecan in Combination with Other Anticancer Therapies in Patients with HER2-Low Metastatic Breast Cancer

**DOI:** 10.1158/1078-0432.CCR-25-0874

**Published:** 2026-01-08

**Authors:** Komal Jhaveri, Sherene Loi, Erika Hamilton, Peter Schmid, Carey K. Anders, Laura Testa, Hans Wildiers, Ling-Ming Tseng, Yen-Shen Lu, Yeon Hee Park, Seock-Ah Im, Shin-Cheh Chen, Robyn R. Young, Caron Lloyd, Magdalena Wrona, Cuihong Zhang, Danielle Carroll, Fabrice André

**Affiliations:** 1 Department of Medicine, Memorial Sloan Kettering Cancer Center, New York, New York.; 2 Weill Cornell Medical College, New York, New York.; 3 Breast Unit, Peter MacCallum Cancer Centre, Melbourne, Australia.; 4 The Sir Peter MacCallum Department of Medical Oncology, The University of Melbourne, Parkville, Australia.; 5 Breast Cancer and Gynecologic Cancer Research, Sarah Cannon Research Institute, Nashville, Tennessee.; 6 Centre for Experimental Cancer Medicine, Barts Cancer Institute, Queen Mary University of London, London, United Kingdom.; 7 Division of Medical Oncology, Duke Cancer Institute, Durham, North Carolina.; 8 Instituto D’Or de Pesquisa e Ensino (IDOR), São Paulo, Brazil.; 9 Department of General Medical Oncology, University Hospitals Leuven, Belgium.; 10 Comprehensive Breast Health Center, Taipei Veterans General Hospital, Taipei, Taiwan.; 11 School of Medicine, National Yang-Ming Chiao Tung University, Taipei, Taiwan.; 12 Department of Oncology, National Taiwan University Hospital, Taipei, Taiwan.; 13 Division of Hematology-Oncology, Department of Medicine, Samsung Medical Center, Sungkyunkwan University School of Medicine, Seoul, Republic of Korea.; 14 Department of Internal Medicine, Cancer Research Institute, Seoul National University Hospital, Seoul, Republic of Korea.; 15 Division of General Surgery, Department of Surgery, Linkou Chang Gung Memorial Hospital, Taoyuan, Taiwan.; 16 The Center for Cancer and Blood Disorders, Fort Worth, Texas.; 17 Clinical Development, Late Development Oncology, Oncology R&D, AstraZeneca, New York, New York.; 18 Late Development Oncology, Global Medicines Development, Oncology R&D, AstraZeneca, Warsaw, Poland.; 19 Biometrics, Late-Stage Development, Oncology R&D, AstraZeneca, Gaithersburg, Maryland.; 20 Translational Medicine, Oncology R&D, AstraZeneca, Cambridge, United Kingdom.; 21 Department of Medical Oncology, Gustave Roussy, Paris-Saclay University, Villejuif, France.

## Abstract

**Purpose:**

Establish the safety, tolerability, and preliminary activity of trastuzumab deruxtecan (T-DXd) in combination with other anticancer therapies in human epidermal growth factor receptor 2 (HER2)–low metastatic breast cancer (mBC).

**Patients and Methods:**

DESTINY-Breast08 was a two-part, open-label, multicenter, phase Ib study. Patients with locally confirmed HER2-low mBC received T-DXd plus capecitabine, durvalumab + paclitaxel, capivasertib, anastrozole, or fulvestrant. Eligibility criteria for hormone receptor status varied across modules and between study parts. Primary objectives were safety/tolerability and determining recommended phase II doses (RP2D); secondary endpoints included objective response rate (ORR; per investigator).

**Results:**

In the dose-finding phase, 37 patients were assigned to a module. RP2Ds were determined for T-DXd plus capecitabine, capivasertib, anastrozole, or fulvestrant. For strategic reasons, T-DXd + durvalumab + paclitaxel was not pursued beyond the dose-finding phase (*n* = 3). In the dose-expansion phase, 101 patients were assigned to a module. For T-DXd + capecitabine, grade ≥3 adverse events (AE) occurred in 55% (11/20) of patients, and the ORR was 60%. For T-DXd + capivasertib, grade ≥3 AEs occurred in 67.5% (27/40) of patients, and the ORR was 60%. For T-DXd + anastrozole, grade ≥3 AEs occurred in 47.6% (10/21) of patients, and the ORR was 71.4%. For T-DXd + fulvestrant, grade ≥3 AEs occurred in 55% (11/20) of patients, and the ORR was 40%. Adjudicated drug-related interstitial lung disease/pneumonitis events were reported for T-DXd + capecitabine (3/20; grade 2, *n* = 2; grade 5, *n* = 1), T-DXd + capivasertib (8/40; all grade ≤2), and T-DXd + fulvestrant (5/20; all grade 2).

**Conclusions:**

Safety results were generally consistent with known individual profiles for T-DXd and combination drugs. T-DXd plus capecitabine, capivasertib, anastrozole, or fulvestrant demonstrated preliminary clinical activity in patients with HER2-low mBC.

**Translational Relevance**

Trastuzumab deruxtecan (T-DXd) is approved for adults with human epidermal growth factor receptor 2 (HER2)–low unresectable/metastatic breast cancer (mBC) who have received prior chemotherapy in the metastatic setting or who have developed disease recurrence during or within 6 months of completing adjuvant chemotherapy, based on the DESTINY-Breast04 trial. T-DXd has also been recently approved for adults with hormone receptor–positive, HER2-low, or HER2-ultralow unresectable/mBC who have progressed on one or more endocrine therapies in the metastatic setting, based on the DESTINY-Breast06 trial. As several studies have suggested upregulation of HER2 protein expression by chemotherapy and endocrine therapy, this study was designed to investigate the potential for other anticancer therapies to increase T-DXd antitumor activity. In this study, we show that safety results were generally consistent with known individual profiles for T-DXd and combination partner drugs. T-DXd plus capecitabine, capivasertib, anastrozole, or fulvestrant demonstrated preliminary clinical activity in patients with HER2-low mBC.

## Introduction

Human epidermal growth factor receptor 2 (HER2)–low breast cancer is an established subset of HER2-negative breast cancer, defined as immunohistochemistry (IHC) 1+ or IHC 2+/*in situ* hybridization–negative (ISH−; ref. 1). Across studies of patients with primary or metastatic breast cancer (mBC), up to approximately 50% have HER2-low tumors (2).

Trastuzumab deruxtecan (T-DXd) is an antibody–drug conjugate (ADC) composed of a HER2-directed antibody, a tetrapeptide-based cleavable linker, and a potent topoisomerase I inhibitor payload (3, 4). In the phase III DESTINY-Breast04 trial, T-DXd demonstrated a significant efficacy benefit compared with physician’s choice of chemotherapy (TPC) among patients with hormone receptor (HR)–positive or HR-negative HER2-low mBC (5). In the HR-positive cohort, median progression-free survival (PFS) was 10.1 months with T-DXd versus 5.4 months with TPC (hazard ratio, 0.51; *P* < 0.001), with a confirmed objective response rate (cORR) of 52.6% versus 16.3%, respectively (5). T-DXd was subsequently approved for the treatment of adult patients with unresectable or metastatic HER2-low breast cancer who have received prior chemotherapy in the metastatic setting or developed disease recurrence during or within 6 months of completing adjuvant chemotherapy (6–8), and the regimen was incorporated into clinical practice guidelines (9, 10).

More recently, in the phase III DESTINY-Breast06 trial, T-DXd demonstrated a significant improvement in PFS versus TPC (hazard ratio, 0.62; *P* < 0.001; median 13.2 vs. 8.1 months), with a cORR of 56.5% versus 32.2%, respectively, in patients with HR-positive, HER2-low mBC who had ≥1 line of endocrine-based therapy but no prior chemotherapy for mBC (11). The results were consistent in an exploratory HER2-ultralow (IHC 0 with membrane staining) cohort (11). Subsequently, T-DXd received approval for adult patients with unresectable or metastatic HR-positive, HER2-low, or HER2-ultralow breast cancer who are not considered suitable for endocrine therapy (ET) as the next line of treatment (6–8).

Preclinical studies suggest that HER2 protein expression may be upregulated by chemotherapy (12), ET-induced modifications, or cross-talk between HER2 and estrogen receptor pathways (13–15). The safety and potentially enhanced efficacy of HER2-directed therapies in combination with chemotherapy or ET have previously been explored, with promising results from phase III clinical trials (16–18).

*Chemotherapy*: Capecitabine is a fluoropyrimidine-based chemotherapy that leads to cytotoxicity via a different mechanism than T-DXd (19) and has shown clinical efficacy in combination with the HER2-directed agents lapatinib (16) and trastuzumab (17) and the topoisomerase I inhibitor irinotecan (with or without bevacizumab; ref. 20). Furthermore, the current standard of care (SOC) in neoadjuvant and metastatic triple-negative breast cancer (TNBC) combines chemotherapy and immune checkpoint inhibitors (21, 22). Like T-DXd, the chemotherapeutic agent paclitaxel promotes an immunogenic tumor microenvironment (23, 24). Combining T-DXd and chemotherapy with or without an immune checkpoint inhibitor (such as durvalumab) may further enhance anticancer activity.

*AKT inhibitors*: The phosphoinositide 3-kinase (PI3K)/protein kinase (AKT) pathway promotes cell proliferation and resistance to apoptosis and is implicated in the development of resistance to chemotherapy and ET through alterations in phosphatidylinositol-4,5-bisphosphate 3-kinase catalytic subunit alpha (*PIK3CA*), AKT serine/threonine protein kinase 1 (*AKT1*), and/or phosphatase and tensin homolog (*PTEN*; refs. 25–29). PI3K inhibition in combination with trastuzumab emtansine (T-DM1) has been demonstrated as tolerable and active in patients with trastuzumab-resistant HER2-positive mBC (30). Capivasertib is a potent, selective inhibitor of all three AKT isoforms (AKT1/2/3; ref. 31); data from the phase III CAPItello-291 study led to the first regulatory approval for capivasertib plus fulvestrant in patients with HR-positive, HER2-negative advanced breast cancer and one or more tumor biomarker alterations (*PIK3CA*, *AKT1*, or *PTEN*) and the inclusion of this treatment option in guidelines (32–34).

*ET*: Anastrozole and fulvestrant are well-established SOC agents in HR-positive breast cancer (21). Cotargeting multiple cytotoxicity pathways by combining ET and HER2-directed therapy has been shown to be beneficial in patients with HR-positive, HER2-positive mBC (18, 35); however, limited data are available for ADCs in combination with ET, regardless of HER2 status. Concurrent ET was permitted in the KATHERINE trial of T-DM1, which is currently an SOC therapy in the residual disease setting for HER2-positive breast cancer (36).

The ongoing phase Ib/2 DESTINY-Breast07 (NCT04538742) and phase Ib/2 BEGONIA (NCT03742102) studies are exploring the antitumor activity of T-DXd in combination with other anticancer agents in patients with HER2-positive mBC and TNBC, respectively. Here, we present the results of DESTINY-Breast08, a phase Ib study designed to establish the safety, tolerability, and preliminary activity of T-DXd in combination with other anticancer agents (capecitabine, durvalumab, paclitaxel, capivasertib, anastrozole, and fulvestrant) that have the potential to increase T-DXd antitumor activity as second- or later-line treatment (dose-finding phase) and first- and second-line treatment (dose-expansion phase) in patients with HER2-low advanced breast cancer/mBC.

## Patients and Methods

### Study design

DESTINY-Breast08 was a two-part, open-label, modular, multicenter, phase Ib study (NCT04556773), conducted between December 17, 2020, and August 16, 2023.

**Figure 1.**
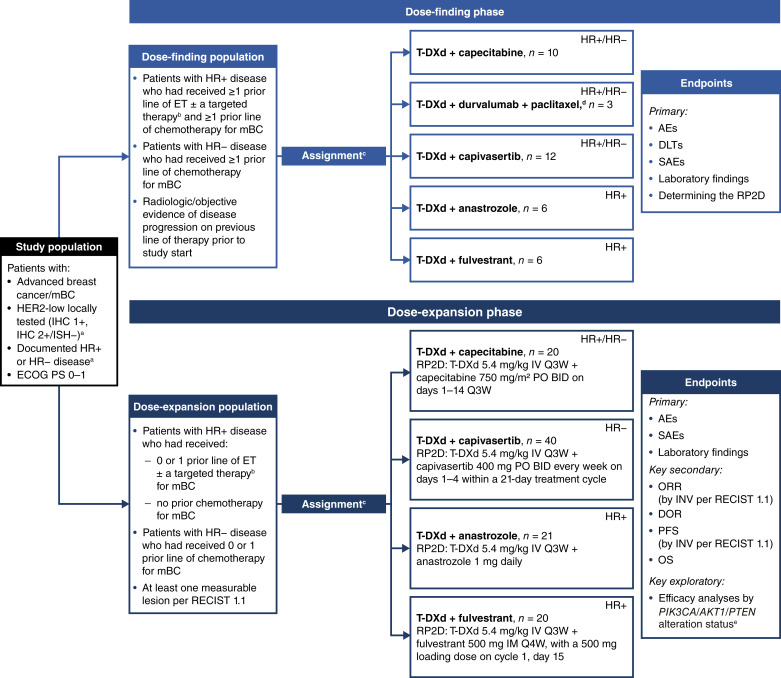
Study design. Abbreviations: AE, adverse event; *AKT1*, AKT serine/threonine protein kinase 1; ASCO/CAP, American Society of Clinical Oncology/College of American Pathologists; BID, twice a day; CDK4/6, cyclin-dependent kinase 4/6; DLT, dose-limiting toxicity; DOR, duration of response; ECOG PS, Eastern Cooperative Oncology Group performance status; ET, endocrine therapy; HER2, human epidermal growth factor receptor 2; HR, hormone receptor; IHC, immunohistochemistry; IM, intramuscularly; INV, investigator; ISH, in situ hybridization; IV, intravenously; mBC, metastatic breast cancer; mTOR, mammalian target of rapamycin; ORR, objective response rate; OS, overall survival; PI3K, phosphoinositide 3-kinase; *PIK3CA*, phosphatidylinositol-4,5-bisphosphate 3-kinase catalytic subunit alpha; PFS, progression-free survival; PO, orally; *PTEN*, phosphatase and tensin homolog; Q3W, once every 3 weeks; Q4W, once every 4 weeks; RECIST 1.1, Response Evaluation Criteria in Solid Tumors version 1.1; RP2D, recommended phase 2 dose; SAE, serious adverse event; T-DXd, trastuzumab deruxtecan. ^a^Per ASCO/CAP guidelines. ^b^Targeted therapies such as CDK4/6, mTOR, or PI3K inhibitors. ^c^Patients were assigned per predefined rules by a central system using interactive response technology, depending on HR status and line of therapy. ^d^The T-DXd + durvalumab + paclitaxel module was not investigated in the dose-expansion phase of the study. ^e^*PIK3CA/AKT1/PTEN* alteration status was a preplanned molecularly defined subgroup of interest in the T-DXd + capivasertib module.

The study comprised a dose-finding phase and a dose-expansion phase; initiation of the dose-expansion phase was dependent on safety findings in the dose-finding phase. Five treatment modules were included: (i) T-DXd + capecitabine, (ii) T-DXd + durvalumab + paclitaxel, (iii) T-DXd + capivasertib, (iv) T-DXd + anastrozole, and (v) T-DXd + fulvestrant (Fig. 1). As the study was open-label, there was no blinding in any of the modules. Patients were enrolled from 37 centers across 10 countries.

The trial protocol was reviewed and approved by an Institutional Review Board prior to study initiation and conducted in accordance with the International Council for Harmonisation Good Clinical Practice guidelines, the Declaration of Helsinki, and local regulations about the conduct of clinical research. All patients provided written informed consent before participation.

### Patient eligibility

Adult patients (≥18 years of age) were required to have locally confirmed HER2-low (IHC 1+ or IHC 2+/ISH−) advanced breast cancer/mBC documented as HR-positive or HR-negative, an Eastern Cooperative Oncology Group performance status of 0 or 1, and radiologic or objective evidence of disease progression on or after the last systemic therapy prior to starting study treatment. Patients with clinically inactive or previously treated brain metastases who were no longer symptomatic and who did not require treatment with corticosteroids or anticonvulsants were eligible for inclusion. Patients were randomly assigned to study modules by central interactive response technology, depending on HR status and prior lines of therapy. Further eligibility criteria are described in Supplementary Information S1.

#### Dose-finding phase

Patients with HR-positive or HR-negative disease were eligible for the T-DXd + capecitabine, T-DXd + durvalumab + paclitaxel, and T-DXd + capivasertib modules. Only patients with HR-positive disease were eligible for the T-DXd + anastrozole and T-DXd + fulvestrant modules. Patients with HR-positive disease must have received at least one prior line of ET with or without a targeted therapy and at least one prior line of chemotherapy for mBC. Those with HR-negative disease must have received at least one prior line of chemotherapy for mBC.

#### Dose-expansion phase

Patients were required to have ≥1 measurable lesion per Response Evaluation Criteria in Solid Tumors version 1.1 (RECIST 1.1) that was not previously irradiated. Patients with HR-positive disease were eligible for the T-DXd + capecitabine, T-DXd + anastrozole, and T-DXd + fulvestrant modules. Patients with HR-negative disease were eligible for the T-DXd + capecitabine, T-DXd + durvalumab + paclitaxel, and T-DXd + capivasertib modules. Patients with HR-positive disease could have received no or one prior line of ET, with or without a targeted therapy (such as cyclin-dependent kinase 4 and 6, mammalian target of rapamycin, or PI3K inhibitors) for mBC; prior chemotherapy in the metastatic setting was exclusionary. Those with HR-negative disease could have received no or one prior line of chemotherapy for mBC in the T-DXd + capecitabine and T-DXd + capivasertib modules; prior chemotherapy for mBC was exclusionary in the T-DXd + durvalumab + paclitaxel module.

For both the dose-finding and dose-expansion phases, capecitabine, anastrozole, and fulvestrant as the most recent anticancer therapy for mBC were exclusionary in the T-DXd + capecitabine, T-DXd + anastrozole, and T-DXd + fulvestrant modules, respectively. Any AKT or PI3K inhibitor as the most recent anticancer therapy for mBC was exclusionary in the T-DXd + capivasertib module. For the dose-finding phase of the T-DXd + durvalumab + paclitaxel module, paclitaxel as the most recent anticancer therapy for mBC was exclusionary.

### Treatment

#### Dose-finding phase

In the T-DXd + capecitabine module, dose level 1 was T-DXd 5.4 mg/kg (intravenously once every 3 weeks) + capecitabine 750 mg/m^2^ (orally twice a day on days 1–14 every 3 weeks). In the T-DXd + durvalumab + paclitaxel module, dose level 1 was T-DXd 5.4 mg/kg (intravenously once every 3 weeks) + durvalumab 1,120 mg (intravenously once every 3 weeks) + paclitaxel 60 mg/m^2^ (intravenously every week within a 21-day treatment cycle). In the T-DXd + capivasertib module, dose level 1 was T-DXd 5.4 mg/kg (intravenously once every 3 weeks) + capivasertib 320 mg (orally twice a day every week on days 1–4 within a 21-day treatment cycle). In the T-DXd + anastrozole module, dose level 1 was T-DXd 5.4 mg/kg (intravenously once every 3 weeks) + anastrozole 1 mg (orally daily). In the T-DXd + fulvestrant module, dose level 1 was T-DXd 5.4 mg/kg (intravenously once every 3 weeks) + fulvestrant 500 mg [intramuscularly every 4 weeks (±1 day)] + fulvestrant 500 mg loading dose (on day 15 of cycle 1). Further information on dose levels for escalation and de-escalation is available in Supplementary Table S1.

#### Dose-expansion phase

The dose-expansion phase used the recommended phase II dose (RP2D) for each combination, as determined in the dose-finding phase of the study.

For both the dose-finding and dose-expansion phases, “on treatment” was defined as the time between the date of the first dose of study treatment and either the date of death or the date of the last dose of study treatment plus the in-survival safety follow-up period. The in-survival follow-up period was defined as 90 days (for T-DXd + durvalumab + paclitaxel) or 47 days (for remaining modules) from the last dose of study treatment.

### Endpoints

#### Dose-finding phase

Primary endpoints were adverse events (AE), serious AEs (SAE), dose-limiting toxicities (DLT), and laboratory findings. AE and SAE collections were coded using standard terms from the Medical Dictionary for Regulatory Activities (MedDRA) version 26.0 and National Cancer Institute Common Terminology Criteria for Adverse Events (NCI CTCAE) version 5.0. If a patient developed radiographic changes potentially consistent with interstitial lung disease (ILD)/pneumonitis or developed an acute onset of new or worsening pulmonary or other related signs/symptoms such as dyspnea (resting or exertional), cough, fever, unexplained fatigue, or a decrease in oxygen saturation, a prompt investigation and appropriate management for ILD/pneumonitis were initiated. Potential cases of ILD/pneumonitis were reviewed by an independent adjudication committee.

A minimum of three evaluable patients were treated through the DLT evaluation period at each dose level. The safety review committee reviewed safety data, including any reported DLTs, from all evaluable patients who received the same dose of treatment. A DLT was defined as any treatment-related AE not attributable to disease or disease-related processes that occurred during the DLT evaluation period and was grade ≥3 according to NCI CTCAE version 5.0, with details and exceptions as defined in Supplementary Table S2. A dose level was considered unsafe if it had an estimated probability of ≥95% of exceeding the 30% target DLT rate.

#### Dose-expansion phase

Primary endpoints were AEs, SAEs, and laboratory findings. Key secondary endpoints included ORR by investigator per RECIST 1.1, duration of response (DOR), PFS by investigator per RECIST 1.1, and overall survival (OS). For the modules containing molecularly defined subgroups of special interest (T-DXd + durvalumab + paclitaxel and T-DXd + capivasertib), there was a target enrollment of approximately 40 patients. All other modules had a target enrollment of approximately 20 patients.

#### *PIK3CA/AKT1/PTEN* alterations

In the T-DXd + capivasertib module, central circulating tumor DNA (ctDNA) collected at baseline was tested for *PIK3CA/AKT1/PTEN* alterations using the Guardant Health OMNI platform. A ctDNA low status indicates that the patient was tested for *PIK3CA/AKT1/PTEN* alterations, but the status could not be determined owing to low shedding; “unknown” signifies that the patient was not tested for *PIK3CA/AKT1/PTEN* alterations.

### Statistical methods

As the study was exploratory in nature, no formal statistical hypothesis testing was performed, and sample sizes were also not determined based on any formal statistical hypothesis. DLT analyses for the dose-finding phase of each module were performed using the DLT-evaluable analysis set, which included all patients who received one full prescribed dose of T-DXd and ≥65% of the prescribed dose of the other study treatment(s) and completed the first treatment cycle or received T-DXd and other study treatment(s) (any amount) and experienced a DLT during the first cycle. Safety analyses for the dose-finding and dose-expansion phases of each module were performed using the safety analysis set, which included all patients who received at least one dose of ≥1 investigational product. In the dose-expansion phase, the planned efficacy analysis set included all patients who received at least one dose of ≥1 investigational product in the dose-expansion phase and had measurable disease at baseline per investigator assessment per RECIST 1.1; of note, the efficacy analysis set corresponded to the safety analysis set for each module.

For the secondary endpoint of ORR, the Clopper–Pearson method was used to estimate the response rate and the 95% confidence interval (CI) for each treatment combination. For PFS, the distribution was estimated using the Kaplan–Meier method, with the corresponding 95% CIs estimated using the Brookmeyer–Crowley method. DOR was reported as calculated from the Kaplan–Meier curves, with medians and their corresponding 95% CIs estimated using the Brookmeyer–Crowley method.

## Results

### Dose-finding phase

Between December 17, 2020, and February 15, 2022, 48 patients were enrolled. Of these, 37 patients were assigned to a module and received treatment, eight patients failed study screening, and three withdrew. At the end of the study period (August 16, 2023), six (16.2%) patients continued to receive study treatment and 32 (86.5%) patients had discontinued T-DXd: 51.4% discontinued due to objective disease progression (RECIST 1.1–defined radiologic progression); 16.2% discontinued due to AE; 13.5% discontinued due to subjective disease progression (global deterioration of health status without objective evidence of RECIST 1.1–defined radiologic progression); and 5.4% discontinued due to patient withdrawal. All patients were female, and the median age was 55 years (range: 35–74). Further information on patient demographics and disposition in the dose-finding phase is reported in Supplementary Table S3.

T-DXd 5.4 mg/kg + capecitabine 750 mg/m^2^ (dose level 1) was received by seven patients, and no DLTs were observed. Subsequently, three more patients received dose level 2 (T-DXd 5.4 mg/kg + capecitabine 1,000 mg/m^2^), of which two patients experienced DLTs: one patient had decreased appetite, fatigue, and nausea (all grade 3), and one patient had colitis (grade 3). Overall safety results and AEs of special interest are reported in Supplementary Tables S4 and S5. Dose level 1 was identified as the RP2D.

T-DXd 5.4 mg/kg + durvalumab 1,120 mg + paclitaxel 60 mg/m^2^ (dose level 1) was received by three patients. No patients experienced DLTs. Initial safety data indicated that further investigation of T-DXd + durvalumab + paclitaxel dose levels or alternative dosing regimens would be required to optimize overall tolerability and determine the RP2D for the triplet combination; however, due to a strategic decision and a change in the development program for T-DXd, further investigation was not pursued.

T-DXd 5.4 mg/kg + capivasertib 320 mg (dose level 1) was received by six patients. A DLT was reported in one patient (grade 4 decreased neutrophil count, recovered at study completion). Six patients received dose level 2 (T-DXd 5.4 mg/kg + capivasertib 400 mg), none of whom experienced a DLT. One nondisease–related death occurred on treatment, due to intracranial hemorrhage not related to T-DXd or capivasertib. Dose level 2 was identified as the RP2D.

T-DXd 5.4 mg/kg + anastrozole 1 mg and T-DXd 5.4 mg/kg + fulvestrant 500 mg (dose level 1, which determined the RP2D) were each received by six patients. No DLT events were reported for either module, and as they were only investigated at a single dose level, dose escalation was not an available option. Across modules, no patients received a de-escalated dose.

Across all modules, changes and grade shifts from baseline in hematology and chemistry parameters were consistent with the respective AEs experienced by patients; there were no clinically important hematology or chemistry abnormalities observed, and no Hy’s law cases were reported. No clinically relevant changes in vital signs, electrocardiogram, or urinalysis values over time were observed at any dose level.

### Dose-expansion phase

#### Overall patient baseline demographics and disposition

Between June 30, 2021, and January 17, 2023, 132 patients were enrolled. Of these, 101 (76.5%) patients were assigned to a module and received treatment; 30 (22.7%) failed study screening; and one (0.8%) withdrew. All patients were female, and the median age was 57 years (range: 29–78; Table 1). At study completion, 37 (36.6%) patients were ongoing study treatment. Across modules, the primary reason for T-DXd discontinuation was objective disease progression (39.6%). All treated patients (*n* = 101) were included in the safety analysis set.

**Table 1.** Patient demographics, baseline characteristics, and disposition (dose-expansion phase).

​	T-DXd + capecitabine (*n* = 20)	T-DXd + capivasertib (*n* = 40)	T-DXd + anastrozole (*n* = 21)	T-DXd + fulvestrant (*n* = 20)
Age, median (range), years	57.5 (36–74)	56 (33–78)	55 (29–75)	65.5 (31–73)
Female, *n* (%)	20 (100)	40 (100)	21 (100)	20 (100)
Race, *n* (%)^a^	​	​	​	​
Asian	10 (50)	18 (45)	11 (52.4)	12 (60)
White	10 (50)	18 (45)	10 (47.6)	7 (35)
Black or African American	0	3 (7.5)	0	1 (5)
HER2 status, *n* (%)	​	​	​	​
IHC 1+	11 (55)	29 (72.5)	16 (76.2)	13 (65)
IHC 2+/ISH−	9 (45)	10 (25)	5 (23.8)	7 (35)
IHC 2+/ISH missing	0	1 (2.5)	0	0
HR status, *n* (%)	​	​	​	​
ER+ and PgR+	9 (45)	0	14 (66.7)	10 (50)
ER+ and PgR−	4 (20)	0	7 (33.3)	9 (45)
ER− and PgR+	1 (5)	0	0	0
ER− and PgR−	6 (30)	39 (97.5)	0	0
PgR missing or ER and PgR missing	0	1 (2.5)^b^	0	1 (5)
ECOG PS, *n* (%)	​	​	​	​
0	10 (50)	30 (75)	12 (57.1)	17 (85)
1	10 (50)	10 (25)	8 (38.1)	3 (15)
2	0	0	1 (4.8)	0
No prior line of therapy for mBC, *n* (%)	6 (30)	22 (55)	7 (33.3)	6 (30)
One prior line for mBC, *n* (%)	14 (70)	18 (45)	14 (66.7)^c^	14 (70)^d^
Patients ongoing treatment at study completion, *n* (%)	7 (35)	17 (42.5)	6 (28.6)	7 (35)
Patients who discontinued both drugs, *n* (%)	13 (65)	23 (57.5)	15 (71.4)	13 (65)
Patients who discontinued T-DXd, *n* (%)	13 (65)	26 (65)	15 (71.4)	16 (80)
Objective disease progression^e^	10 (50)	17 (42.5)	8 (38.1)	5 (25)
Subjective disease progression^e^	0	0	3 (14.3)	2 (10)
Patient decision	2 (10)	2 (5)	0	4 (20)
AE	1 (5)	7 (17.5)	4 (19)	5 (25)
Patients who discontinued combination agent, *n* (%)^f^	15 (75)	28 (70)	15 (71.4)	13 (65)

Abbreviations: AE, adverse event; ECOG PS, Eastern Cooperative Oncology Group performance status; ER, endocrine receptor; HER2, human epidermal growth factor receptor 2; HR, hormone receptor; IHC, immunohistochemistry; ISH, in situ hybridization; mBC, metastatic breast cancer; PgR, progesterone receptor; RECIST 1.1, Response Evaluation Criteria in Solid Tumors version 1; T-DXd, trastuzumab deruxtecan.

a Race for one patient in the T-DXd + capivasertib module was reported as “Other”.

b The patient was confirmed as HR-negative after the first dose.

c All patients received hormonal therapy with targeted therapy.

d Eleven patients received hormonal therapy with targeted therapy, and three received hormonal therapy alone.

e RECIST 1.1–defined radiologic progression was reported as objective disease progression. Subjective disease progression was defined as symptomatic deterioration (global deterioration of health status) without objective evidence of RECIST 1.1–defined radiologic progression.

f Discontinuation of capecitabine, capivasertib, anastrozole, or fulvestrant only (T-DXd treatment was continued).

#### T-DXd + capecitabine

##### Patient disposition

Twenty patients were assigned to T-DXd 5.4 mg/kg + capecitabine 750 mg/m^2^; of these, 14 (70%) patients were HR-positive, and six (30%) were HR-negative (Table 1). A prior line of therapy for mBC was received by nine out of 14 (64.3%) patients with HR-positive disease; four (28.6%) patients had received adjuvant treatment only, and one (7.1%) had no previous treatment (*de novo* disease) at study start. Of the six patients with HR-negative disease, five (83.3%) had received a prior line of therapy for mBC, and one (16.7%) had received adjuvant treatment only. At study completion, seven (35%) patients continued to receive study treatment; the median duration of follow-up (time from first dose to the date of death or date of censoring) was 15.2 months (range: 0.5–17.5).

##### Safety

The median actual treatment duration (total treatment duration, excluding the duration of dose interruptions and delays) was 11.1 months (range: 0.7–16.6) for T-DXd and 7 months (range: 0–16.1) for capecitabine. All patients treated with T-DXd + capecitabine experienced an AE; grade ≥3 AEs occurred in 11 (55%) patients. The most common AEs possibly related to either investigational product (occurring in ≥25% of patients) were nausea (70%, *n* = 14), vomiting (55%, *n* = 11), fatigue (45%, *n* = 9), stomatitis (35%, *n* = 7), decreased appetite (25%, *n* = 5), decreased neutrophil count (25%, *n* = 5), and palmar-plantar erythrodysesthesia (25%, *n* = 5). Two (10%) patients experienced at least one SAE [*n* = 2 (leukocytosis *n* = 1; pneumonitis *n* = 1)]. Ten (50%) patients experienced at least one AE leading to dose reduction of either investigational product, and 15 (75%) patients experienced at least one AE leading to dose interruption. One (5%) and three (15%) patients experienced AEs leading to discontinuation of T-DXd and capecitabine, respectively.

Adjudicated drug-related ILD/pneumonitis events occurred in three (15%) patients [grade 2, *n* = 2 (at study completion, one event had resolved, and one had not resolved); grade 5, *n* = 1]. The adjudicated grade 5 ILD/pneumonitis event was reported in a patient who was >65 years of age and presented without any symptoms 44 days after starting treatment with T-DXd + capecitabine. Radiologic findings indicated a hypersensitivity pneumonitis pattern (grade 2 at onset per investigator, considered grade 3 at onset by the adjudication committee). The event was considered related to T-DXd, per the investigator. Both T-DXd and capecitabine were permanently discontinued, and the patient was hospitalized. There were no signs of infection. The patient received steroid treatment, along with treatment for other non-serious AEs that occurred around the same time as pneumonitis onset; however, pneumonitis was reported as fatal 18 days after discontinuing T-DXd + capecitabine. Key safety outcomes across modules are reported in Table 2; AEs of special interest related to combination agents are reported in Supplementary Table S6.

**Table 2.** Safety summary (dose-expansion phase).

​	T-DXd + capecitabine (*n* = 20)	T-DXd + capivasertib (*n* = 40)	T-DXd + anastrozole (*n* = 21)	T-DXd + fulvestrant (*n* = 20)
Any AE, *n* (%)	20 (100)	40 (100)	20 (95.2)	20 (100)
Grade ≥3	11 (55)	27 (67.5)	10 (47.6)	11 (55)
Any AE possibly related to either drug, *n* (%)^a^	19 (95)	40 (100)	20 (95.2)	20 (100)
Grade ≥3	9 (45)	27 (67.5)	7 (33)	10 (50)
SAEs, *n* (%)	2 (10)	13 (32.5)	4 (19)	4 (20)
Possibly related to either drug^a^	1 (5)	12 (30)	1 (4.8)	1 (5)
AEs leading to dose interruptions of T-DXd, *n* (%)	11 (55)	17 (42.5)	12 (57.1)	9 (45)
AEs leading to dose reduction of T-DXd, *n* (%)	7 (35)	5 (12.5)	6 (28.6)	4 (20)
AEs leading to discontinuation of T-DXd, *n* (%)	1 (5)	7 (17.5)	4 (19)	6 (30)
AEs leading to death, *n* (%)	1 (5)	0	1 (4.8)^b^	0
AESIs related to T-DXd, *n* (%)	​	​	​	​
ILD/pneumonitis events adjudicated as related to T-DXd	3 (15)	8 (20)	0	5 (25)
Grade 1	0	1 (2.5)	0	0
Grade 2	2 (10)	7 (17.5)	0	5 (25)
Grade 5	1 (5)^c^	0	0	0
Left ventricular dysfunction possibly related to T-DXd^a^	0	0	1 (4.8); grade 2	1 (5); grade 2
Median actual treatment duration, months (range)	​	​	​	​
T-DXd/combination drug	11.1 (0.7–16.6)/7 (0–16.1)	6.2 (0.7–12.4)/5.5 (0.2–13.7)	10.4 (2.8–22.2)/11 (1.4–22.4)	6.3 (1.4–21.9)/8.3 (1.8–22.5)

Patients with multiple events in the same category were counted only once in that category. Patients with events in more than one category were counted once in each of those categories.

Abbreviations: AE, adverse event; AESI, AEs of special interest; ILD, interstitial lung disease; SAE, serious adverse event; T-DXd, trastuzumab deruxtecan.

a As assessed by investigator.

b Reported by investigator as related to drug-induced pneumonitis; however, the ILD/pneumonitis event was not considered to be drug-induced by adjudication.

c Adjudicated grade 5 ILD/pneumonitis event details: A patient who was >65 years of age presented without any symptoms 44 days after starting treatment with T-DXd + capecitabine; however, radiologic findings indicated a hypersensitivity pneumonitis pattern, and pneumonitis was diagnosed (grade 2 at onset per investigator, considered grade 3 at onset by the adjudication committee). The investigator considered the pneumonitis event to be related to T-DXd. Both T-DXd and capecitabine were permanently discontinued, and the patient was hospitalized. There were no signs of infection. The patient received prednisolone (1–4 mg/kg daily), along with treatment for other non-serious AEs that occurred around the same time as pneumonitis onset. Pneumonitis was reported as fatal 18 days after discontinuing both T-DXd and capecitabine.

##### Antitumor activity

**Figure 2.**
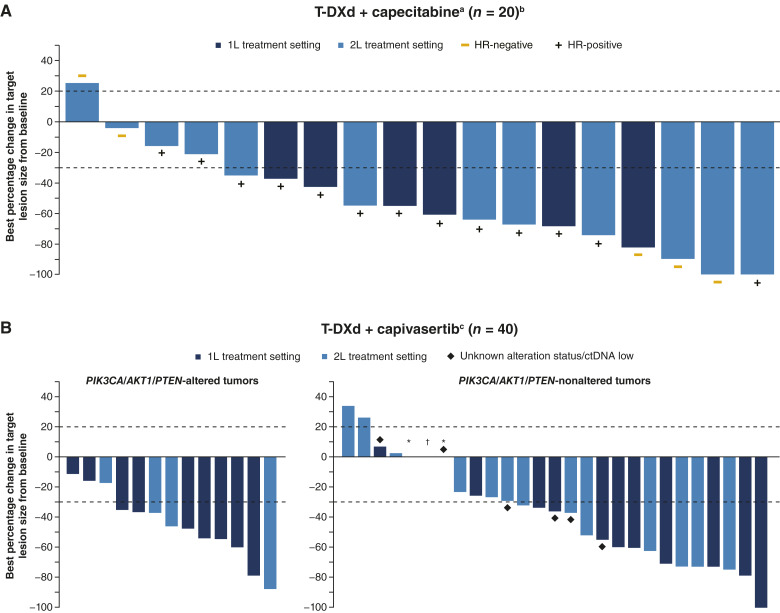
Best percentage change in target lesions from baseline for (**A**) T-DXd + capecitabine and (**B**) T-DXd + capivasertib. 1L, first-line; 2L, second-line; *AKT1*, AKT serine/threonine protein kinase 1; BID, twice daily; ctDNA, circulating tumor DNA; HR, hormone receptor; IV, intravenously; *PIK3CA*, phosphatidylinositol-4,5-bisphosphate 3-kinase catalytic subunit alpha; PO, orally; *PTEN*, phosphatase and tensin homolog; Q1W, every week; Q3W, every 3 weeks; RECIST 1.1, Response Evaluation Criteria in Solid Tumors version 1.1; T-DXd, trastuzumab deruxtecan. T-DXd + capecitabine, n = 20. T-DXd + capivasertib, n = 40; patients with PIK3CA/AKT1/PTEN-altered tumors, n = 13; patients with *PIK3CA/AKT1/PTEN*-non-altered tumors, n = 21; and patients with an unknown/ctDNA low status, n = 6. ^a^T-DXd 5.4 mg/kg IV Q3W + capecitabine 750 mg/m^2^ PO BID on Days 1–14 Q3W. ^b^Two patients (10.0%) did not have any post-baseline RECIST 1.1 data and thus no data were available for best percentage change in target lesion size. ^c^T-DXd 5.4 mg/kg IV Q3W + capivasertib 400 mg PO BID Q1W on Days 1–4 within a 21-day cycle. ^∗^1L treatment setting. †2L treatment setting.

cORR with T-DXd + capecitabine was 60% (12/20; 95% CI, 36.1–80.9); two (10%) patients had a complete response, and 10 (50%) had a partial response (PR) as their best objective response (BOR; Table 3; Fig. 2A). Two (10%) patients did not have complete postbaseline RECIST data. Median DOR (months) was not estimable (NE; *n* = 12; 95% CI, 4.4–NE) at study completion. At study completion, 50% (*n* = 10/20) of patients had experienced a PFS event. Median PFS was 13.4 months (95% CI, 5.5–NE; Supplementary Fig. S1A). Median OS was not mature at study completion; the OS rate at 12 months was 78% (95% CI, 51.5–91.1; Supplementary Fig. S2A). Patient response to treatment for each module is reported in Supplementary Fig. S3.

**Table 3.** Antitumor activity (dose-expansion phase).

​	T-DXd + capecitabine (*n* = 20)	T-DXd + capivasertib (*n* = 40)	T-DXd + anastrozole (*n* = 21)	T-DXd + fulvestrant (*n* = 20)
Median duration of follow-up, months (range)	15.2 (0.5–17.5)	8.6 (1.2–14.9)	20.2 (4.9–24.8)	15.2 (2.2–22.6)
cORR, % (95% CI)	60 (36.1–80.9)	60 (43.3–75.1)	71.4 (47.8–88.7)	40 (19.1–64)
BOR, *n* (%)	​	​	​	​
Complete response	2 (10)	0	0	0
Partial response	10 (50)	24 (60)	15 (71.4)	8 (40)
Stable disease	5 (25)	12 (30)	6 (28.6)	11 (55)
Unconfirmed complete or partial response^a^	2 (10)	2 (5)	1 (4.8)	2 (10)
Stable disease ≥5 weeks	3 (15)	10 (25)	5 (23.8)	9 (45)
Progressive disease	1 (5)	4 (10)	0	1 (5)
Not evaluable	2 (10)	0	0	0
Median DOR, months (95% CI)^b^^,^^c^^,^^d^	NE (4.4–NE)	7.1 (5–NE)	9.8 (6.7–NE)	NE (4.1–NE)
Total PFS events, n (%)	10 (50)	18 (45)	14 (66.7)	7 (35)
Median PFS, months (95% CI)^b^^,^^c^	13.4 (5.5–NE)	9 (5.6–NE)	13.4 (8.5–19.4)	NE (5.6–NE)
PFS at 6 months, % (95% CI)^b^	66.7 (40.4–83.4)	65.5 (48–78.4)	80.7 (56.3–92.3)	75.3 (46.4–90)
PFS at 12 months, % (95% CI)^b^	55.6 (30.5–74.8)	33.9 (12.7–56.6)	50.4 (27.5–69.5)	52.7 (25–74.4)
Total OS events, *n* (%)	5 (25)	4 (10)	6 (28.6)	3 (15)
Median OS, months (95% CI)^b^^,^^c^^,^^d^	NE (14.8–NE)	NE (13.5–NE)	NE (11.7–NE)	NE (NE–NE)
OS at 6 months, % (95% CI)^b^	94.7 (68.1–99.2)	94.8 (80.8–98.7)	95.2 (70.7–99.3)	100 (100–100)
OS at 12 months, % (95% CI)^b^	78 (51.5–91.1)	92 (77.2–97.4)	71.4 (47.2–86)	88.2 (60.6–96.9)

a Includes patients with a complete or partial response with no confirmation assessment performed or with a confirmation assessment performed, but response not confirmed.

b Calculated using the Kaplan–Meier technique.

c CI derived based on the Brookmeyer–Crowley method.

d NE signifies that median DOR/OS was not reached for these patients at the time of study completion.

#### T-DXd + capivasertib

##### Patient disposition

Forty patients were assigned to T-DXd 5.4 mg/kg + capivasertib 400 mg; of these, 39 (97.5%) were confirmed as HR-negative, and HR data were missing for one (2.5%) patient (Table 1). At baseline, *PIK3CA/AKT1/PTEN*-altered tumors were confirmed in 32.5% (*n* = 13/40) of patients, and *PIK3CA/AKT1/PTEN*-nonaltered tumors were confirmed in 52.5% (*n* = 21/40) of patients. In 5% (*n* = 2/40) and 10% (*n* = 4/40) of patients, an unknown or ctDNA-low status was reported, respectively. Eighteen (45%) patients had previously received a first-line treatment for mBC; 19 (47.5%) patients had received adjuvant treatment only; and three (7.5%) had *de novo* disease at study start. At study completion, 17 (42.5%) patients continued to receive study treatment; the median duration of follow-up was 8.6 months (range: 1.2–14.9).

##### Safety

Median actual treatment duration was 6.2 months (range: 0.7–12.4) for T-DXd and 5.5 months (range: 0.2–13.7) for capivasertib. All patients treated with T-DXd + capivasertib experienced an AE; grade ≥3 AEs occurred in 27 (67.5%) patients (Table 2). The most common AEs possibly related to either investigational product were nausea (92.5%, *n* = 37), diarrhea (77.5%, *n* = 31), vomiting (52.5%, *n* = 21), fatigue (45%, *n* = 18), alopecia (37.5%, *n* = 15), anemia (27.5%, *n* = 11), and decreased appetite (27.5%, *n* = 11). Thirteen (32.5%) patients experienced at least one SAE (diarrhea, *n* = 2; *Pneumocystis jirovecii* pneumonia, *n* = 2; pneumonitis, *n* = 2; anemia, *n* = 1; dehydration, *n* = 1; diabetes mellitus, *n* = 1; herpes zoster, *n* = 1; nausea, *n* = 1; peripheral neuropathy, *n* = 1; pleural effusion, *n* = 1; pneumonia, *n* = 1; viral pneumonia, *n* = 1; septic shock, *n* = 1). Twenty-one (52.5%) patients experienced at least one AE that led to a dose reduction of either investigational product, and 28 (70%) patients experienced at least one AE leading to a dose interruption. AEs led to discontinuation of T-DXd and capivasertib in seven (17.5%) and nine (22.5%) patients, respectively.

Adjudicated drug-related ILD/pneumonitis events occurred in eight (20%) patients receiving T-DXd + capivasertib (grade 1, *n* = 1; grade 2, *n* = 7); at study completion, three cases had resolved, and five had not resolved. Capivasertib-related hyperglycemia occurred in eight (20%) patients (highest CTCAE grade reported: grade 1, *n* = 3; grade 2, *n* = 2; grade 3, *n* = 3); at study completion, four cases had resolved, two were resolving, and two had not resolved (Supplementary Table S6). Capivasertib-related rash AEs occurred in seven (17.5%) patients (grade 1, *n* = 2; grade 2, *n* = 2; grade 3, *n* = 3); at study completion, six cases had resolved, and one had not resolved (Supplementary Table S6).

##### Antitumor activity

cORR with T-DXd + capivasertib was 60% (*n* = 24/40; 95% CI, 43.3–75.1), all of whom had a PR as their BOR (Table 3; Fig. 2B). Median DOR was 7.1 months (*n* = 24; 95% CI, 5–NE) at study completion. At study completion, 45% (*n* = 18/40) of patients had experienced a PFS event. Median PFS was 9 months (95% CI, 5.6–NE; Supplementary Fig. S1B). Median OS was not mature at study completion; the 12-month OS rate was 92% (95% CI, 77.2–97.4; Supplementary Fig. S2B).

cORR was 76.9% (*n* = 10/13; 95% CI, 46.2–95) in patients with *PIK3CA/AKT1/PTEN*-altered tumors and 52.4% (*n* = 11/21; 95% CI, 29.8–74.3) in patients with *PIK3CA/AKT1/PTEN*-nonaltered tumors. In patients with an unknown status and ctDNA low status, cORR was 50% (*n* = 1/2 and *n* = 2/4, respectively). Median DOR was 7.1 months (*n* = 2/10; 95% CI, 4.2–NE) in patients with *PIK3CA/AKT1/PTEN*-altered tumors and 5.7 months (*n* = 5/11; 95% CI, 3–NE) in patients with *PIK3CA/AKT1/PTEN*-nonaltered tumors. In patients with an unknown status and ctDNA low status, median DOR was 5 months (*n* = 1/1) and NE (*n* = 1/2), respectively. In patients with *PIK3CA/AKT1/PTEN*-altered tumors, there was a total of two PFS events (*n* = 2/13, 15.4%); median PFS (months) was NE (95% CI, 8.4–NE). In patients with *PIK3CA/AKT1/PTEN*-nonaltered tumors, there was a total of 12 PFS events (*n* = 12/21, 57.1%); median PFS was 6.8 months (95% CI, 4.2–NE). There were two PFS events in patients with an unknown status (*n* = 2/2, 100%) and two PFS events in patients with a ctDNA low status (*n* = 2/4, 50%); median PFS was 3.7 months and NE, respectively.

#### T-DXd + anastrozole

##### Patient disposition

Twenty-one patients were assigned to receive T-DXd 5.4 mg/kg + anastrozole 1 mg. All patients had HR-positive disease; 14 (66.7%) had previously received ET with targeted therapy as first-line mBC treatment, two (9.5%) had received adjuvant ET only, and five (23.8%) had *de novo* disease at study start (Table 1). At study completion, six (28.6%) patients were continuing to receive the combination; the median duration of follow-up was 20.2 months (range: 4.9–24.8).

##### Safety

Median actual treatment duration was 10.4 months (range: 2.8–22.2) for T-DXd and 11 months (range: 1.4–22.4) for anastrozole. Twenty (95.2%) patients experienced an AE, and 10 had grade ≥3 AEs (47.6%; Table 2). The most common AEs possibly related to either investigational product were nausea (66.7%, *n* = 14), alopecia (42.9%, *n* = 9), fatigue (38.1%, *n* = 8), decreased appetite (33.3%, *n* = 7), and vomiting (28.6%, *n* = 6). Four (19%) patients experienced at least one SAE (ascites, *n* = 1; endocarditis, *n* = 1; fall, *n* = 1; hyperammonemia, *n* = 1; pneumonitis, *n* = 1). Six (28.6%) patients experienced at least one AE leading to dose reduction of either investigational product, and 12 (57.1%) patients experienced at least one AE leading to dose interruption. AEs led to discontinuation of T-DXd and anastrozole in four (19%) and two (9.5%) patients, respectively. No adjudicated drug-related ILD/pneumonitis events were reported. One (4.8%) patient experienced grade 2 left ventricular dysfunction (resolved at study completion).

##### Antitumor activity

**Figure 3.**
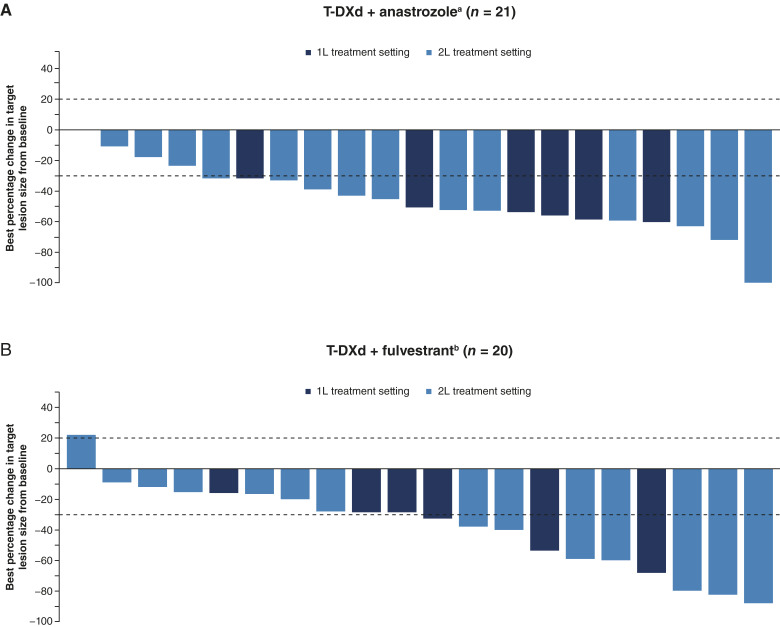
Best percentage change in target lesions from baseline for (**A**) T-DXd + anastrozole and (**B**) T-DXd + fulvestrant. 1L, first-line; 2L, second-line; IM, intramuscularly; IV, intravenously; OD, once daily; PO, orally; Q3W, every 3 weeks; Q4W, every 4 weeks; T-DXd, trastuzumab deruxtecan. ^a^T-DXd 5.4 mg/kg IV Q3W + anastrozole 1 mg PO OD. ^b^T-DXd 5.4 mg/kg IV Q3W + fulvestrant 500 mg IM Q4W with 500-mg loading dose on Day 15 of Cycle 1.

cORR with T-DXd + anastrozole was 71.4% (*n* = 15/21; 95% CI, 47.8–88.7), all of whom had a PR as their BOR (Table 3; Fig. 3A). Median DOR was 9.8 months (*n* = 15; 95% CI, 6.7–NE) at study completion. At study completion, 66.7% (*n* = 14/21) of patients had experienced a PFS event. Median PFS was 13.4 months (95% CI, 8.5–19.4; Supplementary Fig. S1C). Median OS was not mature at study completion; the 12-month OS rate was 71.4% (95% CI, 47.2–86; Supplementary Fig. S2C).

#### T-DXd + fulvestrant

##### Patient disposition

Twenty patients were assigned to T-DXd 5.4 mg/kg + fulvestrant 500 mg. All patients had HR-positive breast cancer, 14 (70%) had previously received ET as first-line treatment for mBC (with targeted therapy, *n* = 11; et alone, *n* = 3), three (15%) had received adjuvant ET only, and three (15%) had *de novo* disease at study start (Table 1). At study completion, seven (35%) patients continued to receive study treatment. The median duration of follow-up was 15.2 months (range: 2.2–22.6).

##### Safety

Median actual treatment duration was 6.31 months (range: 1.4–21.9) for T-DXd and 8.31 months (range: 1.8–22.5) for fulvestrant. All patients treated experienced an AE; grade ≥3 AEs occurred in 11 (55%) patients (Table 2). The most common AEs possibly related to either investigational product were nausea (95%, *n* = 19), alopecia (50%, *n* = 10), decreased appetite (50%, *n* = 10), and vomiting (35%, *n* = 7). SAEs were reported for four (20%) patients (ascites, *n* = 1; atrioventricular block, *n* = 1; ILD/pneumonitis, *n* = 1; urinary tract infection, *n* = 1). Four patients experienced at least one AE leading to dose reduction of either investigational product, and 11 (55%) patients experienced at least one AE leading to dose interruption. Six (30%) patients had AEs leading to the discontinuation of T-DXd; no AEs led to the discontinuation of fulvestrant.

Adjudicated drug-related ILD/pneumonitis events occurred in five (25%) patients (all grade 2); at study completion, two cases had resolved, one was resolving, and two had not resolved. A *post hoc* analysis investigating potential causes found no apparent trend; however, patients seemed to have more potential risk factors compared with other modules. Four of the five patients had at least one of the following potential risk factors: advanced age (>65 years, *n* = 3), moderate renal impairment at baseline (*n* = 1), prior thoracic radiation (*n* = 1), and time since initial diagnosis >4 years (*n* = 3). One grade 2 left ventricular dysfunction event was reported (resolved at study completion).

##### Antitumor activity

cORR with T-DXd + fulvestrant was 40% (*n* = 8/20; 95% CI, 19.1–64), all of whom had a PR as their BOR (Table 3; Fig. 3B). Median DOR (months) was NE (*n* = 8; 95% CI, 4.1–NE) at study completion. Of note, three (15%) patients withdrew consent and discontinued study treatment before disease progression. At study completion, 35% (*n* = 7/20) of patients had experienced a PFS event. Median PFS (months) was NE (95% CI, 5.6–NE; Supplementary Fig. S1D). Median OS was not mature at study completion; the 12-month OS rate was 88.2% (95% CI, 60.6–96.9; Supplementary Fig. S2D).

## Discussion

DESTINY-Breast08 was designed to investigate the safety of T-DXd in combination with chemotherapy, an AKT inhibitor, and ETs while preliminarily exploring the potential of these combinations to improve clinical outcomes in HER2-low mBC.

Safety results observed in this study were generally consistent with the known individual safety profiles for T-DXd and each combination drug. Initial safety data obtained in the dose-finding phase indicated that further investigation of T-DXd + durvalumab + paclitaxel dose levels or alternative dosing regimens would be required for optimization of tolerability; however, further investigation of this triplet combination was not pursued. During the dose-expansion phase of the remaining modules, most AEs were mild or moderate in severity and were manageable via dose modifications and routine clinical practice. The rate of AEs of grade 3 or higher and SAEs was numerically higher with T-DXd + capivasertib; however, conclusions should be made with caution owing to the small datasets.

Overall, adjudicated drug-related ILD/pneumonitis events were reported in 15.8% of patients (*n* = 16/101). Based on the event with the highest CTCAE grade reported for each patient, most adjudicated ILD/pneumonitis events were grade 1 or 2 (93.8%, *n* = 15/16) and manageable following dose modification and established ILD management guidelines, including monitoring signs and symptoms of ILD/pneumonitis (e.g., cough, fever, dyspnea) and proactively managing events with early intervention (such as dose modification, corticosteroid treatment, and supportive care). These data are in line with a pooled analysis of nine T-DXd monotherapy studies across multiple tumor types, in which the incidence of adjudicated drug-related ILD/pneumonitis events was 15.4%, of which 77.4% were of a low CTCAE grade (worst AE grade of 1 or 2; ref. 37). One grade 5 adjudicated ILD/pneumonitis event occurred in the T-DXd + capecitabine module. *Post hoc* analysis indicated no apparent trend relating to the incidence of adjudicated drug-related ILD/pneumonitis with T-DXd + fulvestrant (25%). Although the risk profile for developing ILD/pneumonitis in patients treated with T-DXd is currently not well defined, patients in the T-DXd + fulvestrant module seemed to meet more of the factors considered potential risks compared with other modules [age (>65 years), renal impairment, exposure to radiation, time since initial diagnosis (>4 years)]. Studies are ongoing to further understand the etiology of ILD/pneumonitis and to identify potential biomarkers.

Results of the dose-expansion phase demonstrated the antitumor activity of T-DXd in combination with capecitabine, capivasertib, anastrozole, or fulvestrant in terms of ORR, DOR, and PFS. Treatment with T-DXd + capecitabine resulted in a cORR of 60%, a median PFS of 13.4 months, and a 12-month OS rate of 78%. For the overall population of the T-DXd + capivasertib module, we report a cORR of 60%, a median PFS of 9 months, and a 12-month OS rate of 92%. The cORR, median PFS, and 12-month OS rates with T-DXd + anastrozole were 71.4%, 13.4 months, and 71.4%, while the cORR, median PFS, and 12-month OS rates with T-DXd + fulvestrant were 40%, NE, and 88.2%, respectively. However, the small datasets in this study limit the interpretation of the efficacy results. The relative contribution of each individual agent or the potential for a synergistic effect as a combination cannot be discerned as the study design did not comprise a T-DXd monotherapy module.

A further limitation of the study design is that statistical comparative analyses between prespecified subgroups with or without *PIK3CA/AKT1/PTEN* alterations in the T-DXd + capivasertib module were not planned; additionally, subgroup analyses based on HR status in the T-DXd + capecitabine module were not planned. A further limitation is the absence of the HER2-ultralow subgroup (IHC 0 with membrane staining; refs. 1, 38), following the consistent benefit observed with T-DXd across both HER2-low and HER2-ultralow populations in the DESTINY-Breast06 trial (11). T-DXd combinations were only investigated as first-line therapy in the metastatic setting in a small number of patients (*n* = 41), limiting the interpretation of results in this setting. Representativeness of the study population is shown in Supplementary Table S7. At present, further studies investigating these combinations in patients with HER2-low mBC are not planned; nevertheless, the safety data reported here are reassuring for the ongoing DESTINY-Breast05 (NCT04622319) and DESTINY-Breast09 (NCT04784715) studies, which both allow concurrent use of ET with T-DXd in patients with HER2-positive breast cancer.

In conclusion, no new safety signals were identified for T-DXd or the combination agents during this study. T-DXd in combination with capecitabine, capivasertib, anastrozole, or fulvestrant demonstrated preliminary clinical activity in patients with HER2-low mBC.

## Supplementary Material

Supplementary Data 1 Supplementary Data

## Data Availability

Data underlying the findings described in this article may be obtained in accordance with AstraZeneca’s data-sharing policy described at https://www.astrazenecaclinicaltrials.com/our-transparency-commitments/. Data for studies directly listed on Vivli can be requested through Vivli at www.vivli.org. Data for studies not listed on Vivli can be requested through Vivli at https://vivli.org/members/enquiries-about-studies-not-listed-on-the-vivli-platform/. AstraZeneca’s Vivli member page is also available, outlining further details: https://vivli.org/ourmember/astrazeneca/.
