# Supplementary material for: DESTINY-Breast08: A Phase Ib Study of Trastuzumab Deruxtecan in Combination with Other Anticancer Therapies in Patients with HER2-Low Metastatic Breast Cancer
Source: Clin Cancer Res. 2026 Jan 8;32(6):1046–58. doi: 10.1158/1078-0432.CCR-25-0874 (PMC13016200; doi:10.1158/1078-0432.CCR-25-0874)
Supplement: Supplementary Data 1 — Supplementary Data [file ccr-25-0874_supplementary_data_1_suppds1.docx]

# SUPPLEMENTARY INFORMATION

Contents

[SUPPLEMENTARY INFORMATION 1](#_Toc198108018)

[**Table S1.** Dose levels (dose-finding phase). 2](#_Toc198108019)

[**Table S2.** Dose-limiting toxicity definitions and exceptions. 3](#_Toc198108020)

[**Table S3.** Patient demographics, baseline characteristics, and disposition (dose-finding phase). 4](#_Toc198108021)

[**Table S4.** Safety summary (dose-finding phase). 5](#_Toc198108022)

[**Table S5.** Adverse events of special interest (dose-finding phase). 6](#_Toc198108023)

[**Table S6.** Adverse events of special interest related to combination agents (dose-expansion phase). 7](#_Toc198108024)

[**Table S7.** Representativeness of study participants. 8](#_Toc198108025)

[**Figure S1.** Progression-free survival with a) T-DXd + capecitabine, b) T-DXd + capivasertib, c) T-DXd + anastrozole, or d) T-DXd + fulvestrant. 9](#_Toc198108026)

[**Figure S2.** Kaplan-Meier analysis of overall survival in patients receiving a) T-DXd + capecitabine, b) T-DXd + capivasertib, c) T-DXd + anastrozole, or d) T-DXd + fulvestrant. 11](#_Toc198108027)

[**Figure S3.** Patient response for a) T-DXd + capecitabine, b) T-DXd + capivasertib, c) T-DXd + anastrozole, and d) T-DXd + fulvestrant throughout the study period. 13](#_Toc198108028)

[**Supplementary Information S1.** Study inclusion and exclusion criteria. 15](#_Toc198108029)

[Inclusion criteria 15](#_Toc198108030)

[Exclusion criteria 16](#_Toc198108031)

[**Supplementary References** 17](#_Toc198108032)

## **Table S1.** Dose levels (dose-finding phase).

|  | **Dose level (1)** | **Dose level (2)** | **Dose level (−1)** |
| --- | --- | --- | --- |
| T-DXd | 5.4 mg/kg IV Q3W | 5.4 mg/kg IV Q3W | 4.4 mg/kg IV Q3W |
| Capecitabine | 750 mg/m^2^ PO BID, Days 1–14, Q3W | 1000 mg/m^2^ PO BID, Days 1–14, Q3W | 750 mg/m^2^ PO BID, Days 1–14, Q3W |

|  | **Dose level (1)** | **Dose level (2)** | **Dose level (−1)** |
| --- | --- | --- | --- |
| T-DXd | 5.4 mg/kg IV Q3W | 5.4 mg/kg IV Q3W | 4.4 mg/kg IV Q3W |
| Durvalumab | 1120 mg IV Q3W | 1120 mg IV Q3W | 1120 mg IV Q3W |
| Paclitaxel | 60 mg/m^2^ IV Q1W | 80 mg/m^2^ IV Q1W | 60 mg/m^2^ IV Q1W |

|  | **Dose level (1)** | **Dose level (2)** | **Dose level (−1)** | **Dose level (−2)** |
| --- | --- | --- | --- | --- |
| T-DXd | 5.4 mg/kg IV Q3W | 5.4 mg/kg IV Q3W | 5.4 mg/kg IV Q3W | 4.4 mg/kg IV Q3W |
| Capivasertib | 320 mg PO BID | 400 mg PO BID | 200 mg PO BID | 200 mg PO BID |
|  | All capivasertib doses on an intermittent weekly dosing schedule (4 days on, 3 days off).  Patients were dosed every week on Days 1 to 4 within a 21-day treatment cycle. | | | |

|  | **Dose level (1)** | **Dose level (−1)** |
| --- | --- | --- |
| T-DXd | 5.4 mg/kg IV Q3W | 4.4 mg/kg IV Q3W |
| Anastrozole | 1 mg PO daily | 1 mg PO daily |

|  | **Dose level (1)** | **Dose level (−1)** |
| --- | --- | --- |
| T-DXd | 5.4 mg/kg IV Q3W | 4.4 mg/kg IV Q3W |
| Fulvestrant | 500 mg IM Q4W (± 1 day) + 500-mg loading dose on Day 15 of Cycle 1 | 500 mg IM Q4W (± 1 day) + 500-mg loading dose on Day 15 of Cycle 1 |

Abbreviations: BID, twice daily; IM, intramuscularly; IV, intravenously; PO, orally; Q1W, every week; Q3W, every 3 weeks; Q4W, every 4 weeks; T-DXd, trastuzumab deruxtecan.

## **Table S2.** Dose-limiting toxicity definitions and exceptions.

|  | **Definitions** | **Exceptions** |
| --- | --- | --- |
| Hematologic toxicities | - - Grade 4 neutrophil count decreased lasting >7 days   - Febrile neutropenia   - Grade 4 anemia   - Grade 4 platelet count decreased   - Grade 3 platelet count decreased lasting >7 days   - Grade 3 platelet count decreased with clinically significant hemorrhage   - Grade 4 lymphocyte count decreased lasting ≥14 days |  |
| Hepatic organ toxicities | - Grade 4 AST or ALT increased - AST or ALT >3 × ULN, if accompanied by grade >2 blood bilirubin increased - In patients without liver metastases, AST or ALT >5 × ULN lasting >3 days - In patients with liver metastases, AST or ALT >5 × ULN lasting >3 days, if the baseline level was ≤3 × ULN - In patients with liver metastases, AST or ALT >8 × ULN lasting >3 days, if the baseline level was >3 × ULN |  |
| Non-hematologic, non-hepatic toxicities | - Symptomatic congestive heart failure - LVEF decline to <40%, or >20% drop from baseline - Other grade ≥3 non-hematologic, non-hepatic toxicities | - Grade 3 fatigue lasting <7 days - Grade 3 nausea, vomiting, diarrhea, or anorexia that has resolved to grade ≤2 within 3 days - Isolated laboratory findings not associated with signs or symptoms, including but not limited to, grade 3/4 alkaline phosphatase increased, hyperuricemia, serum amylase increased, lipase increased, and grade 3 hyponatremia lasting <72 hours developed from normal or grade 1 at baseline - Grade 3 lymphocyte count decreased - AEs that were clearly not related to study treatment (e.g., car accidents) per investigator’s assessment were not considered DLTs |

Abbreviations: AE, adverse event; ALT, alanine transaminase; AST, aspartate transferase; DLT, dose-limiting toxicity; LVEF, left ventricular ejection fraction; ULN, upper limit of normal.

## **Table S3.** Patient demographics, baseline characteristics, and disposition (dose-finding phase).

|  | **T-DXd + capecitabine  (*n* = 10)^a^** | **T-DXd + durvalumab  + paclitaxel (*n* = 3)^a^** | **T-DXd + capivasertib (*n* = 12)^a^** | **T-DXd + anastrozole  (*n* = 6)^a^** | **T-DXd + fulvestrant  (*n* = 6)^a^** |
| --- | --- | --- | --- | --- | --- |
| Age, median (range), years | 56.0 (35.0–64.0) | 52.0 (42.0–55.0) | 55.5 (39.0–73.0) | 57.0 (47.0–71.0) | 58.0 (46.0–74.0) |
| Female, *n* (%) | 10 (100.0) | 3 (100.0) | 12 (100.0) | 6 (100.0) | 6 (100.0) |
| Race, *n* (%) |  |  |  |  |  |
| Asian | 4 (40.0) | 2 (66.7) | 5 (41.7) | 6 (100.0) | 4 (66.7) |
| White | 4 (40.0) | 0 | 4 (33.3) | 0 | 2 (33.3) |
| Black or African American | 1 (10.0) | 1 (33.3) | 0 | 0 | 0 |
| Not reported | 1 (10.0) | 0 | 3 (25.0) | 0 | 0 |
| HER2 status, *n* (%) |  |  |  |  |  |
| IHC 1+ | 7 (70.0) | 2 (66.7) | 7 (58.3) | 3 (50.0) | 2 (33.3) |
| IHC 2+/ISH − | 3 (30.0) | 1 (33.3) | 5 (41.7) | 3 (50.0) | 4 (66.7) |
| HR status, *n* (%) |  |  |  |  |  |
| ER+ and PR+ | 3 (30.0) | 1 (33.3) | 8 (66.7) | 2 (33.3) | 1 (16.7) |
| ER+ and PR− | 2 (20.0) | 1 (33.3) | 2 (16.7) | 4 (66.7) | 5 (83.3) |
| ER− and PR+ | 1 (10.0) | 0 | 0 | 0 | 0 |
| ER− and PR− | 4 (40.0) | 1 (33.3) | 2 (16.7) | 0 | 0 |
| ECOG PS, *n* (%) |  |  |  |  |  |
| 0 | 3 (30.0) | 2 (66.7) | 6 (50.0) | 5 (83.3) | 4 (66.7) |
| 1 | 7 (70.0) | 1 (33.3) | 5 (41.7) | 1 (16.7) | 2 (33.3) |
| Missing | 0 | 0 | 1 (8.3) | 0 | 0 |
| Patients ongoing treatment at study completion, *n* (%) | 1 (10.0) | 0 | 2 (16.7) | 0 | 3 (50.0) |
| Patients who discontinued all agents, *n* (%) | 9 (90.0) | 3 (100) | 10 (83.3) | 6 (100) | 3 (50.0) |
| Patients who discontinued T-DXd, *n* (%) | 9 (90.0) | 3 (100) | 10 (83.3) | 6 (100) | 4 (66.7) |
| Objective disease progression^b^ | 5 (50.0) | 2 (66.7) | 5 (41.7) | 4 (66.7) | 3 (50.0) |
| Subjective disease progression^b^ | 2 (20.0) | 1 (33.3) | 0 | 2 (33.3) | 0 |
| Subject decision | 2 (20.0) | 0 | 0 | 0 | 0 |
| AE | 0 | 0 | 5 (41.7) | 0 | 1 (16.7) |
| Patients who discontinued the combination agent(s), *n* (%)^c^ | 9 (90.0) | 3 (100.0) / 3 (100.0) | 10 (83.3) | 6 (100) | 3 (50.0) |

Abbreviations: AE, adverse event; ECOG PS, Eastern Cooperative Oncology Group performance status; ER, endocrine receptor; HER2, human epidermal growth factor receptor 2; HR, hormone receptor; IHC, immunohistochemistry; ISH, in situ hybridization; PR, progesterone receptor; RECIST 1.1, Response Evaluation Criteria in Solid Tumors version 1.1; T-DXd, trastuzumab deruxtecan. ^a^Safety outcomes reported across both dose levels investigated for the T-DXd + capecitabine and T-DXd + capivasertib modules (T-DXd 5.4 mg/kg + capecitabine 750 mg/m^2^ [dose level 1; *n* = 7] and T-DXd 5.4 mg/kg + capecitabine 1000 mg/m^2^ [dose level 2; *n* = 3]; T-DXd 5.4 mg/kg + capivasertib 320 mg [dose level 1; *n* = 6] and T-DXd 5.4 mg/kg + capivasertib 400 mg [dose level 2; *n* = 6]). Only one dose level was investigated for the following modules: T-DXd 5.4 mg/kg + durvalumab 1120 mg + paclitaxel 60 mg/m^2^, T-DXd 5.4 mg/kg + anastrozole 1 mg, and T-DXd 5.4 mg/kg + fulvestrant 500 mg.
^b^RECIST 1.1-defined radiological progression was reported as objective disease progression. Subjective disease progression was defined as symptomatic deterioration (global deterioration of health status) without objective evidence of RECIST 1.1-defined radiological progression.
^c^Discontinuation of capecitabine, durvalumab/paclitaxel, capivasertib, anastrozole or fulvestrant only (T-DXd treatment was continued).

## **Table S4.** Safety summary (dose-finding phase).

|  | **T-DXd + capecitabine  (*n* = 10)^a^** | **T-DXd + durvalumab  + paclitaxel (*n* = 3)^a^** | **T-DXd + capivasertib (*n* = 12)^a^** | **T-DXd + anastrozole  (*n* = 6)^a^** | **T-DXd + fulvestrant  (*n* = 6)^a^** |
| --- | --- | --- | --- | --- | --- |
| Any AEs, *n* (%) | 10 (100.0) | 3 (100.0) | 12 (100.0) | 6 (100.0) | 6 (100.0) |
| Grade ≥3 | 7 (70.0) | 3 (100.0) | 9 (75.0) | 3 (50.0) | 4 (66.7) |
| Any AEs possibly related to either drug, *n* (%)^b^ | 10 (100.0) | 3 (100.0) | 12 (100.0) | 6 (100.0) | 6 (100.0) |
| Grade ≥3 | 7 (70.0) | 3 (100.0) | 7 (58.3) | 3 (50.0) | 3 (50.0) |
| SAEs, *n* (%) | 4 (40.0) | 1 (33.3) | 5 (41.7) | 2 (33.3) | 3 (50.0) |
| Possibly related to either drug^b^ | 3 (30.0) | 1 (33.3) | 2 (16.7) | 0 | 1 (16.7) |
| AEs leading to dose interruptions of T-DXd, *n* (%) | 5 (50.0) | 2 (66.7) | 7 (58.3) | 5 (83.3) | 4 (66.7) |
| AEs leading to dose reduction of T-DXd, *n* (%) | 4 (40.0) | 3 (100.0) | 1 (8.3) | 0 | 3 (50.0) |
| AEs leading to discontinuation of T-DXd, *n* (%) | 0 | 0 | 5 (41.7) | 0 | 1 (16.7) |
| AEs leading to death, *n* (%) | 0 | 0 | 1 (8.3)^c^ | 0 | 0 |
| Median actual treatment duration, months (range) |  |  |  |  |  |
| T-DXd (dose level 1) | 4.9 (1.4–20.1) | 8.4 (4.2–20.5) | 10.4 (2.7–27.2) | 7.7 (2.8–16.0) | 20.1 (6.9–28.1) |
| Combination drug(s) (dose level 1) | 4.9 (0.9– 19.9) | 7.0 (4.2–20.5) /  6.2 (1.2–11.9) | 10.7 (1.8– 28.7) | 8.0 (2.4– 17.7) | 24.6 (8.3–26.5) |
| T-DXd (dose level 2) | 2.7 (0.7–6.3) | – | 5.5 (1.4–17.1) | – | – |
| Combination drug (dose level 2) | 0.9 (0.3–1.5) | – | 5.5 (2.5–16.8) | – | – |

Abbreviations: AE, adverse event; SAE, serious adverse event; T-DXd, trastuzumab deruxtecan. ^a^Safety outcomes reported across both dose levels investigated for the T-DXd + capecitabine and T-DXd + capivasertib modules (T-DXd 5.4 mg/kg + capecitabine 750 mg/m^2^ [dose level 1] and T-DXd 5.4 mg/kg + capecitabine 1000 mg/m^2^ [dose level 2], T-DXd 5.4 mg/kg + capivasertib 320 mg [dose level 1] and T-DXd 5.4 mg/kg + capivasertib 400 mg [dose level 2]). Only one dose level was investigated for the following modules: T-DXd 5.4 mg/kg + durvalumab 1120 mg + paclitaxel 60 mg/m^2^, T-DXd 5.4 mg/kg + anastrozole 1 mg, and T-DXd 5.4 mg/kg + fulvestrant 500 mg.
^b^As assessed by investigator.
^c^Primary cause of death: intracranial hemorrhage not reported as possibly related to either study drug by investigator. Patients with multiple events in the same category were counted only once in that category. Patients with events in more than one category were counted once in each of those categories.

## **Table S5.** Adverse events of special interest (dose-finding phase).

|  | **T-DXd + capecitabine  (*n* = 10)^a^** | **T-DXd + durvalumab  + paclitaxel (*n* = 3)^a^** | **T-DXd + capivasertib  (*n* = 12)^a^** | **T-DXd + anastrozole  (*n* = 6)^a^** | **T-DXd + fulvestrant  (*n* = 6)^a^** |
| --- | --- | --- | --- | --- | --- |
| AESIs, n (%) |  |  |  |  |  |
| ILD/pneumonitis events adjudicated as related to T-DXd | 0 | 0 | 5 (41.7) | 2 (33.3) | 2 (33.3) |
| Grade ≤2 | 0 | 0 | 3 (25.0) | 1 (16.7) | 1 (16.7) |
| Grade 3 | 0 | 0 | 2 (16.7) | 0 | 1 (16.7) |
| Grade 5 | 0 | 0 | 0 | 1 (16.7)^b^ | 0 |
| Left ventricular dysfunction possibly related to T-DXd^c^ | 0 | 0 | 1 (8.3); grade 2 | 0 | 1 (16.7); grade 2 |
| Diarrhea/colitis possibly related to durvalumab^c^ | – | 1 (33.3); grade 3 | – | – | – |
| Hyperthyroid event possibly related to durvalumab^c^ | – | 1 (33.3); grade 2 | – | – | – |
| Pancreatic event possibly related to durvalumab^c,d^ | – | 1 (33.3); grade 2 | – | – | – |
| Hyperglycemia possibly related to capivasertib^c^ | – | – | 1 (8.3); grade 2 | – | – |
| Rash AE possibly related to capivasertib^c^ | – | – | 2 (16.7); grade 1 | – | – |

Abbreviations: AE, adverse event; AESI, adverse event of special interest; ILD, interstitial lung disease; T-DXd, trastuzumab deruxtecan. ^a^Safety outcomes reported across both dose levels investigated for the T-DXd + capecitabine and T-DXd + capivasertib modules (T-DXd 5.4 mg/kg + capecitabine 750 mg/m^2^ [dose level 1] and T-DXd 5.4 mg/kg + capecitabine 1000 mg/m^2^ [dose level 2], T-DXd 5.4 mg/kg + capivasertib 320 mg [dose level 1] and T-DXd 5.4 mg/kg + capivasertib 400 mg [dose level 2]). Only one dose level was investigated for the following modules: T-DXd 5.4 mg/kg + durvalumab 1120 mg + paclitaxel 60 mg/m^2^, T-DXd 5.4 mg/kg + anastrozole 1 mg, and T-DXd 5.4 mg/kg + fulvestrant 500 mg with 500-mg loading dose.
^b^Adjudicated grade 5 ILD/pneumonitis event details: lung inflammation with productive cough was reported in a patient who was <65 years of age 120 days after starting T-DXd + anastrozole. Lung inflammation was not considered ILD/pneumonitis by investigator but was considered related to T-DXd. There were no signs of infection. T-DXd was interrupted, and the patient received prednisolone 5 mg (to be taken as needed) for 21 days. T-DXd and anastrozole were discontinued permanently due to objective disease progression (on Days 205 and 211, respectively), and the patient died ~2 weeks later. Death was considered to be related to underlying cancer by investigator (primary cause: breast cancer; secondary cause: oxygen saturation decreased); however, the AESI was adjudicated as ILD/pneumonitis by committee (grade 1 at onset, maximum grade 5).
^c^As assessed by investigator.
^d^Grouped term for increased amylase and lipase levels. Patients with multiple events in the same category were counted only once in that category. Patients with events in more than one category were counted once in each of those categories.

## **Table S6.** Adverse events of special interest related to combination agents (dose-expansion phase).

|  | **T-DXd + capecitabine  (*n* = 20)** | **T-DXd + capivasertib  (*n* = 40)** | **T-DXd + anastrozole  (*n* = 21)** | **T-DXd + fulvestrant  (*n* = 20)** |
| --- | --- | --- | --- | --- |
| Hyperglycemia possibly related to capivasertib^a^ | – | 8 (20.0) | – | – |
| Grade 1 | – | 3 (7.5) | – | – |
| Grade 2 | – | 2 (5.0) | – | – |
| Grade 3 | – | 3 (7.5) | – | – |
| Rash AE possibly related to capivasertib^a,b^ | – | 7 (17.5) | – | – |
| Grade 1 | – | 2 (5.0) | – | – |
| Grade 2 | – | 2 (5.0) | – | – |
| Grade 3 | – | 3 (7.5) | – | – |

Abbreviations: AE, adverse event; AESI, adverse event of special interest; T-DXd, trastuzumab deruxtecan.

Patients with multiple events in the same category were counted only once in that category. Patients with events in more than one category were counted once in each of those categories.

No AESIs related to capecitabine, anastrozole, or fulvestrant were reported. ^a^As assessed by investigator.
^b^Grouped term.

## **Table S7.** Representativeness of study participants.

| Cancer type(s) / subtype(s) / stage(s) / condition | HER2-low advanced/mBC |
| --- | --- |
| Considerations related to: |  |
| Sex | In the US, breast cancer cases in women account for approximately 99% of all breast cancer cases and deaths (1). Accordingly, retrospective analyses have demonstrated the incidence of HER2-low breast cancer in men to be less than 1% (2,3). |
| Age | In women, the median age at breast cancer diagnosis is 62 years and the incidence rate increases until approximately 70 years of age (1). In a HER2-low breast cancer population, the median age at diagnosis was 59 years (2); in a second study, 87% of patients were reported to be ≥50 years of age (3). |
| Race/ethnicity | The incidence rate of breast cancer (per 100,000) is highest among White (137.9) and Black women (131.3), with lower rates reported in AIAN (123.6), AAPI (108.3), and Hispanic (104.1) women across the US. Despite the lower incidence, mortality rates (per 100,000) are higher among Black (26.8) and AIAN (20.5) women than White women (19.4) (1). Across all races/ethnicities, HER2-negative breast cancer is the predominant subtype, accounting for 76–82% of all cases (1). |
| Geography | Female breast cancer incidence rates (per 100,000) are highest in France, Australia / New Zealand, Northern America, and Northern Europe (>90), and are more than three times higher than in South-Central Asia and Middle Africa (<30) (4). |
| Other considerations | Compared with other subtypes, women diagnosed with HR-positive, HER2-negative breast cancer are more likely to drink alcohol, use hormone replacement therapy (in the five years prior to diagnosis), and be older, and women with HR-negative, HER2-negative breast cancer are more likely to be younger and overweight (5–9). |
| Overall representativeness of this study | The difference in breast cancer incidence rates between females and males is represented in this study, with 100% of participants being female.  The median age of study participants was similar to the reported median age at diagnosis (55 years [dose-finding phase] and 57 years [dose-expansion phase] vs 62 years) (1).  Asian and White individuals comprised the majority of study participants (dose-finding phase: 57% and 27%, respectively; dose-expansion phase: 51% and 45%, respectively), while Black or African American individuals were underrepresented (~5% in each phase).  Participants were recruited from across the world. In the dose-finding phase, participants were from Asia (57%), Australia (16%), North America (16%), and Europe (11%). In the dose-expansion phase, participants were from Asia (56%), North America (15%), Brazil (11%), Australia (9%), and Europe (9%). |

Abbreviations: AAPI, Asian American / Pacific Islander; AIAN, American Indian / Alaska Native; HER2, human epidermal growth factor receptor 2; HR, hormone receptor; mBC, metastatic breast cancer.

## **Figure S1.** Progression-free survival with a) T-DXd + capecitabine, b) T-DXd + capivasertib, c) T-DXd + anastrozole, or d) T-DXd + fulvestrant.


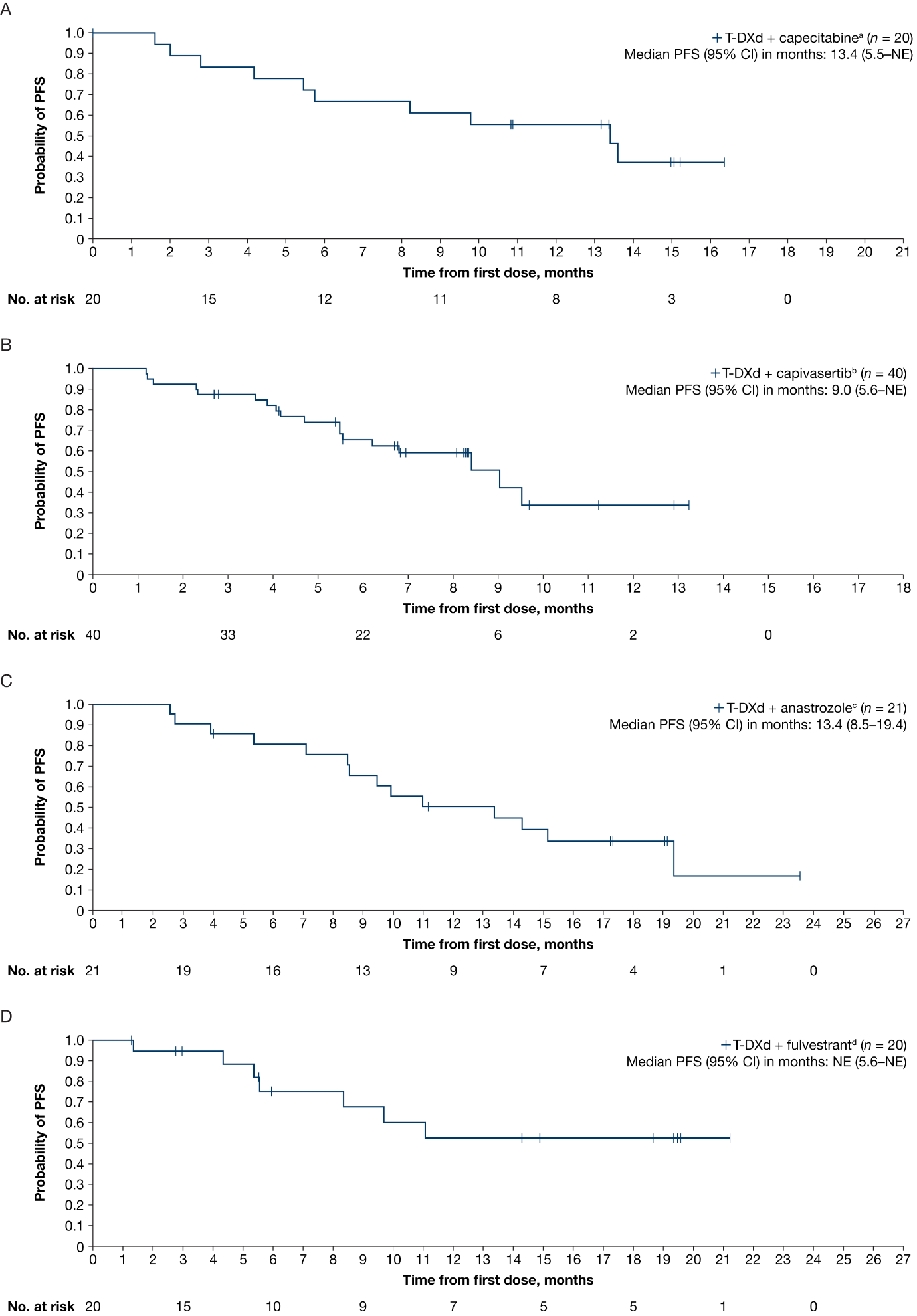


Abbreviations: BID, twice daily; CI, confidence interval; IM, intramuscularly; IV, intravenously; NE, not estimable; OD, once daily; PFS, progression-free survival; PO, orally; Q1W, every week; Q3W, every 3 weeks; Q4W, every 4 weeks; T-DXd, trastuzumab deruxtecan.
T-DXd + capecitabine, *n* = 20. T-DXd + capivasertib, *n* = 40. T-DXd + anastrozole, *n* = 21. T-DXd + fulvestrant, *n* = 20. ^a^T-DXd 5.4 mg/kg IV Q3W + capecitabine 750 mg/m^2^ PO BID on Days 1–14 Q3W.
^b^T-DXd 5.4 mg/kg IV Q3W + capivasertib 400 mg PO BID Q1W on Days 1–4 within a 21-day cycle.
^c^T-DXd 5.4 mg/kg IV Q3W + anastrozole 1 mg PO OD.
^d^T-DXd 5.4 mg/kg IV Q3W + fulvestrant 500 mg IM Q4W with 500-mg loading dose on Day 15 of Cycle 1.

## **Figure S2.** Kaplan-Meier analysis of overall survival in patients receiving a) T-DXd + capecitabine, b) T-DXd + capivasertib, c) T-DXd + anastrozole, or d) T-DXd + fulvestrant.


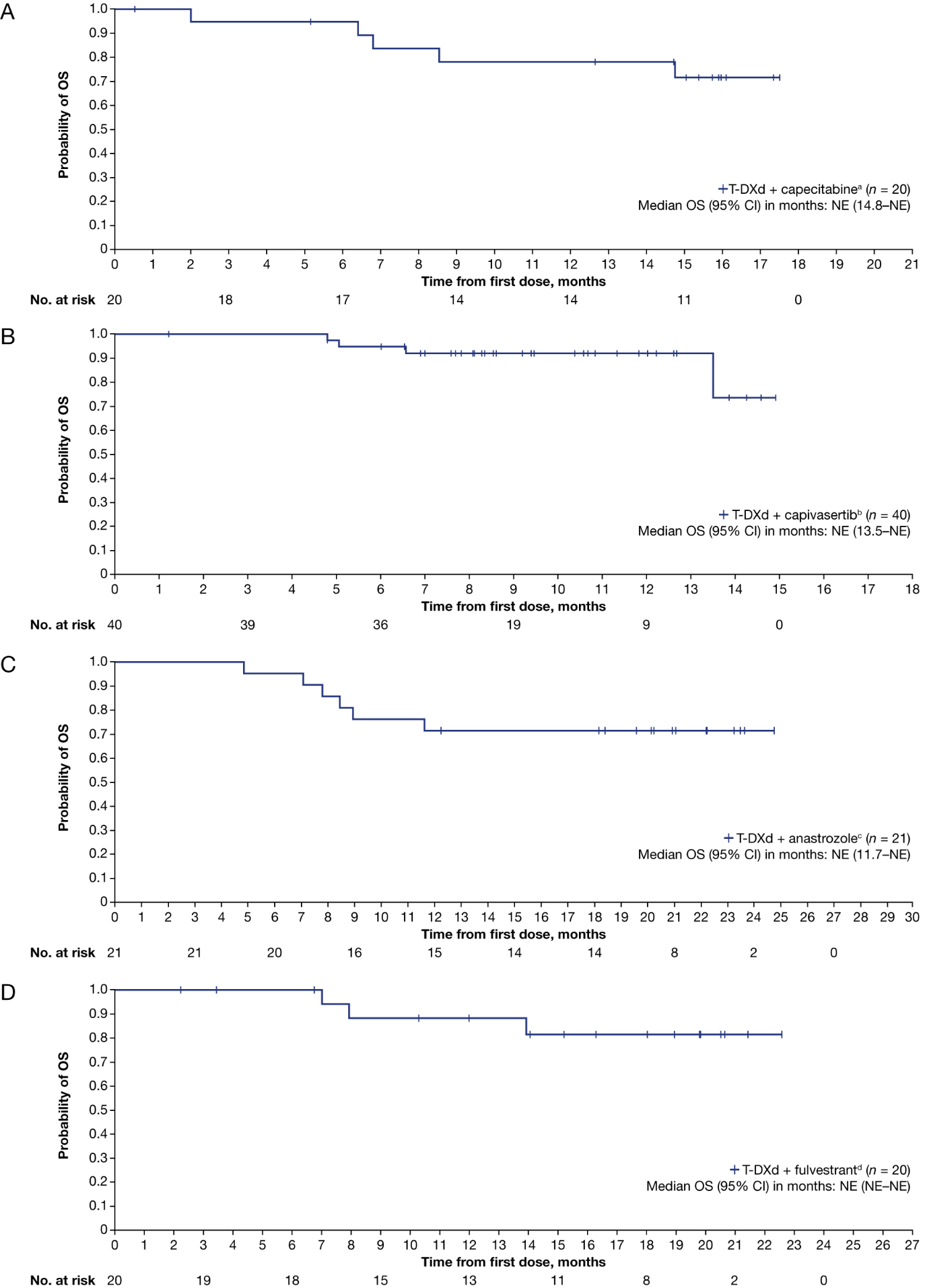


Abbreviations: BID, twice daily; CI, confidence interval; IM, intramuscularly; IV, intravenously; NE, not estimable; OD, once daily; OS, overall survival; PO, orally; Q1W, every week; Q3W, every 3 weeks; Q4W, every 4 weeks; T-DXd, trastuzumab deruxtecan.
T-DXd + capecitabine, *n* = 20. T-DXd + capivasertib, *n* = 40. T-DXd + anastrozole, *n* = 21. T-DXd + fulvestrant, *n* = 20. ^a^T-DXd 5.4 mg/kg IV Q3W + capecitabine 750 mg/m^2^ PO BID on Days 1–14 Q3W.
^b^T-DXd 5.4 mg/kg IV Q3W + capivasertib 400 mg PO BID Q1W on Days 1–4 within a 21-day cycle.
^c^T-DXd 5.4 mg/kg IV Q3W + anastrozole 1 mg PO OD.
^d^T-DXd 5.4 mg/kg IV Q3W + fulvestrant 500 mg IM Q4W with 500-mg loading dose on Day 15 of Cycle 1.

## **Figure S3.** Patient response for a) T-DXd + capecitabine, b) T-DXd + capivasertib, c) T-DXd + anastrozole, and d) T-DXd + fulvestrant throughout the study period.


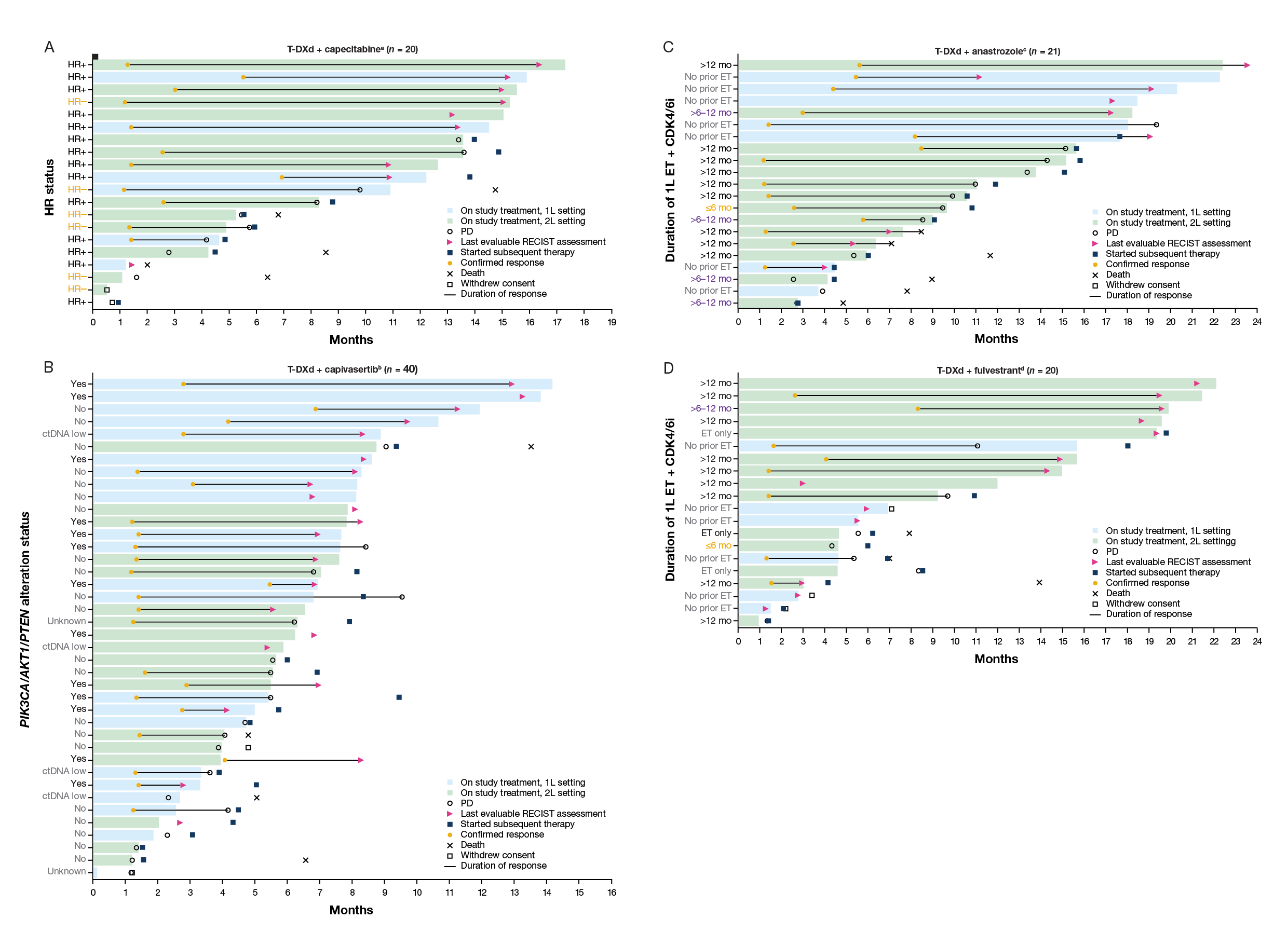


Abbreviations: 1L, first-line; 2L, second-line; *AKT1*, AKT serine/threonine protein kinase 1; BID, twice daily; CDK4/6i, cyclin-dependent kinase 4/6 inhibitor; ctDNA, circulating tumor DNA; ET, endocrine therapy; HR, hormone receptor; IM, intramuscularly; IV, intravenously; mo, months; OD, once daily; PD, disease progression; *PIK3CA*, phosphatidylinositol-4,5-bisphosphate 3-kinase catalytic subunit alpha; PO, orally; *PTEN*, phosphatase and tensin homolog; Q1W, every week; Q3W, every 3 weeks; Q4W, every 4 weeks; RECIST 1.1, Response Evaluation Criteria in Solid Tumors version 1.1; T-DXd, trastuzumab deruxtecan.
T-DXd + capecitabine, *n* = 20. T-DXd + capivasertib, *n* = 40; patients with *PIK3CA/AKT1/PTEN*-altered tumors, *n* = 13; patients with *PIK3CA/AKT1/PTEN*-non-altered tumors, *n* = 21; and patients with an unknown / ctDNA low status, *n* = 6. T-DXd + anastrozole, *n* = 21. T-DXd + fulvestrant, *n* = 20.

^a^T-DXd 5.4 mg/kg IV Q3W + capecitabine 750 mg/m^2^ PO BID on Days 1–14 Q3W.
^b^T-DXd 5.4 mg/kg IV Q3W + capivasertib 400 mg PO BID Q1W on Days 1–4 within a 21-day cycle.
^c^T-DXd 5.4 mg/kg IV Q3W + anastrozole 1 mg PO OD.
^d^T-DXd 5.4 mg/kg IV Q3W + fulvestrant 500 mg IM Q4W with 500-mg loading dose on Day 15 of Cycle 1.

## **Supplementary Information S1.** Study inclusion and exclusion criteria.

### Inclusion criteria

Patients were eligible to be included in the study only if all of the following criteria applied. Eligible patients were adults who were able to provide informed consent and who had pathologically documented advanced or metastatic breast cancer (mBC) that was documented as hormone receptor (HR)-positive (either endocrine receptor and/or progesterone receptor positive [ER or PR ≥1%]) or HR-negative (ER and PR negative [ER and PR <1%]) per American Society of Clinical Oncology / College of American Pathologists (ASCO/CAP) guidelines, with a history of human epidermal growth factor receptor 2 (HER2)-low expression, defined as immunohistochemistry (IHC) 2+/in situ hybridization–negative (ISH−) or IHC 1+ (ISH− or untested) with a validated assay. Previous diagnosis of HER2-positive (IHC 3+ or ISH+) disease was exclusionary.

Patients must have had an adequate tumor tissue sample available and an Eastern Cooperative Oncology Group performance status (ECOG PS) of 0 or 1. Radiologic or objective evidence of disease progression on or after the last systemic therapy prior to starting study treatment, and at least one measurable lesion that was not previously irradiated and could be accurately assessed at baseline by CT (computed tomography) or MRI (magnetic resonance imaging) and was suitable for repeated assessment per Response Evaluation Criteria in Solid Tumors version 1.1 (RECIST 1.1) were required. In the dose-finding phase, patients with HR-positive disease must have received at least one prior line of endocrine therapy (ET) ± a targeted therapy and at least one prior line of chemotherapy for mBC. Those with HR-negative disease must have received at least one prior line of chemotherapy for mBC. In the dose-expansion phase, patients with HR-positive disease could have received no or one prior line of ET ± a targeted therapy for mBC; prior chemotherapy in the metastatic setting was exclusionary. Those with HR-negative disease could have received no or one prior line of chemotherapy for mBC.

Patients must have had left ventricular ejection fraction ≥50% within 28 days before enrollment, and adequate organ and bone marrow function within 14 days before treatment assignment. Study inclusion required adequate treatment washout period(s) before enrollment, and a minimum life expectancy of 12 weeks at screening.

Female patients were required to have evidence of postmenopausal status or a negative serum pregnancy test if they were of childbearing potential and were sexually active with a non-sterilized male partner. Female patients of childbearing potential were required to use at least one highly effective method of contraception. Donation, or retrieval for personal use, of ova from the time of screening and throughout the study treatment period, and for at least 7 months after the final study drug administration, was not allowed.

### Exclusion criteria

Patients were excluded from the study if they had uncontrolled intercurrent illness, uncontrolled or significant cardiovascular disease, or uncontrolled infection requiring treatment at screening. Patients with lung-specific intercurrent clinically significant illnesses, or any autoimmune, connective tissue, or inflammatory disorders with documented, or suspected pulmonary involvement at the time of screening were not eligible for the study. Prior pneumonectomy, presence of spinal cord compression, or clinically active central nervous system metastases (defined as untreated and symptomatic, or requiring therapy corticosteroids or anticonvulsants to control associated symptoms) were exclusionary. Active primary immunodeficiency, known human immunodeficiency virus infection, or active hepatitis B (known positive HBsAg result) or C infection were also exclusionary.

Patients receiving concurrent chemotherapy, investigational product, biologic, or hormonal therapy for cancer treatment were ineligible for the study. Patients, if enrolled, were not to receive live vaccine within 30 days prior to the first dose of T-DXd, during the study and up to 30 days after the last dose of investigation product. Unresolved toxicities from previous anticancer therapy, prior treatment with an antibody-drug conjugate that comprised an exatecan derivative and known allergy or hypersensitivity to study treatment or any of the study drug excipients were also exclusionary.

Patients were not eligible for inclusion in the study if they had involvement in the planning and/or conduct of the study, if they were judged by investigator as unlikely to comply with study procedures, restrictions, and requirements, if they were ineligible for any of the combination therapies, had a history of allogeneic organ transplantation, were pregnant or breastfeeding, or had undergone major surgery (excluding placement of vascular access) within 4 weeks of the first dose of study treatment.

## **Supplementary References**

1. Giaquinto AN, Sung H, Newman LA, Freedman RA, Smith RA, Star J, *et al.* Breast cancer statistics 2024. *CA Cancer J Clin* **2024**;74:477–95.

2. Schettini F, Chic N, Brasó-Maristany F, Paré L, Pascual T, Conte B, *et al.* Clinical, pathological, and PAM50 gene expression features of HER2-low breast cancer. *NPJ Breast Cancer* **2021**;7:1.

3. Baez-Navarro X, van Bockstal MR, Andrinopoulou E-R, van Deurzen CHM. HER2-low breast cancer: incidence, clinicopathologic features, and survival outcomes from real-world data of a large nationwide cohort. *Mod Pathol* **2023**;36:100087.

4. Bray F, Laversanne M, Sung H, Ferlay J, Siegel RL, Soerjomataram I, *et al.* Global cancer statistics 2022: GLOBOCAN estimates of incidence and mortality worldwide for 36 cancers in 185 countries. *CA Cancer J Clin* **2024**;74:229–63.

5. Clarke CA, Keegan TMH, Yang J, Press DJ, Kurian AW, Patel AH, *et al*. Age-specific incidence of breast cancer subtypes: understanding the black-white crossover. *J Natl Cancer Inst* **2012**;104:1094–101.

6. Jenkins EO, Deal AM, Anders CK, Prat A, Perou CM, Carey LA, *et al*. Age-specific changes in intrinsic breast cancer subtypes: A focus on older women. *Oncologist* **2014**;19:1076–83.

7. Gago-Dominguez M, Castelao JE, Gude F, Fernandez MP, Aguado-Barrera ME, Ponte SM, et al. Alcohol and breast cancer tumor subtypes in a Spanish cohort. *Spingerplus* **2016**;5

8. Gomes KAL, de Araújo Jerônimo AF, Guimarães CMC, de Oliveira Ramos R, Dos Santos Andrade LS, Weller M. *Cancer Epidemiol* **2022**;78

9. Rosenberg V, Bareket-Samish A, Chodick G, Siegelmann-Danieli N. *Int J Womens Health* **2021**;13:1207–16
